# Supplementary material for: Low-dose IL-2 reduces IL-21+ T cell frequency and induces anti-inflammatory gene expression in type 1 diabetes
Source: Nat Commun. 2022 Nov 28;13:7324. doi: 10.1038/s41467-022-34162-3 (PMC9705541; doi:10.1038/s41467-022-34162-3)
Supplement: Supplementary file 1 — Supplementary Information [file 41467_2022_34162_MOESM1_ESM.pdf]

Low-dose IL-2 reduces IL-21$^+$ T cell frequency and induces anti-inflammatory gene expression in type 1 diabetes

Jia-Yuan Zhang[1], Fiona Hamey[1], Dominik Trzupek[1], Marius Mickunas[2], Mercede Lee[1], Leila Godfrey[1], Jennie H.M. Yang[2], Marcin L Pekalski[1], Jane Kennet[3,4], Frank Waldron-Lynch[5], Mark L. Evans[3,4], Timothy I. M. Tree[2], Linda S. Wicker[1], John A. Todd[1] and Ricardo C. Ferreira[1]

[1] JDRF/Wellcome Diabetes and Inflammation Laboratory, Wellcome Centre for Human Genetics, Nuffield Department of Medicine, NIHR Oxford Biomedical Research Centre, University of Oxford, Oxford, United Kingdom.

[2] Department of Immunobiology, King's College London, School of Immunology and Microbial Sciences, London, United Kingdom.

[3] Wellcome-MRC Institute of Metabolic Science, Metabolic Research Laboratories, University of Cambridge, Cambridge, United Kingdom.

[4] National Institute for Health Research Cambridge Biomedical Research Centre, Addenbrooke's Biomedical Campus, Cambridge, United Kingdom.

[5] Vertex Pharmaceuticals, Vertex Cell & Gene Therapies, Boston, Massachusetts, United States of America.

Supplementary Figures 1-15

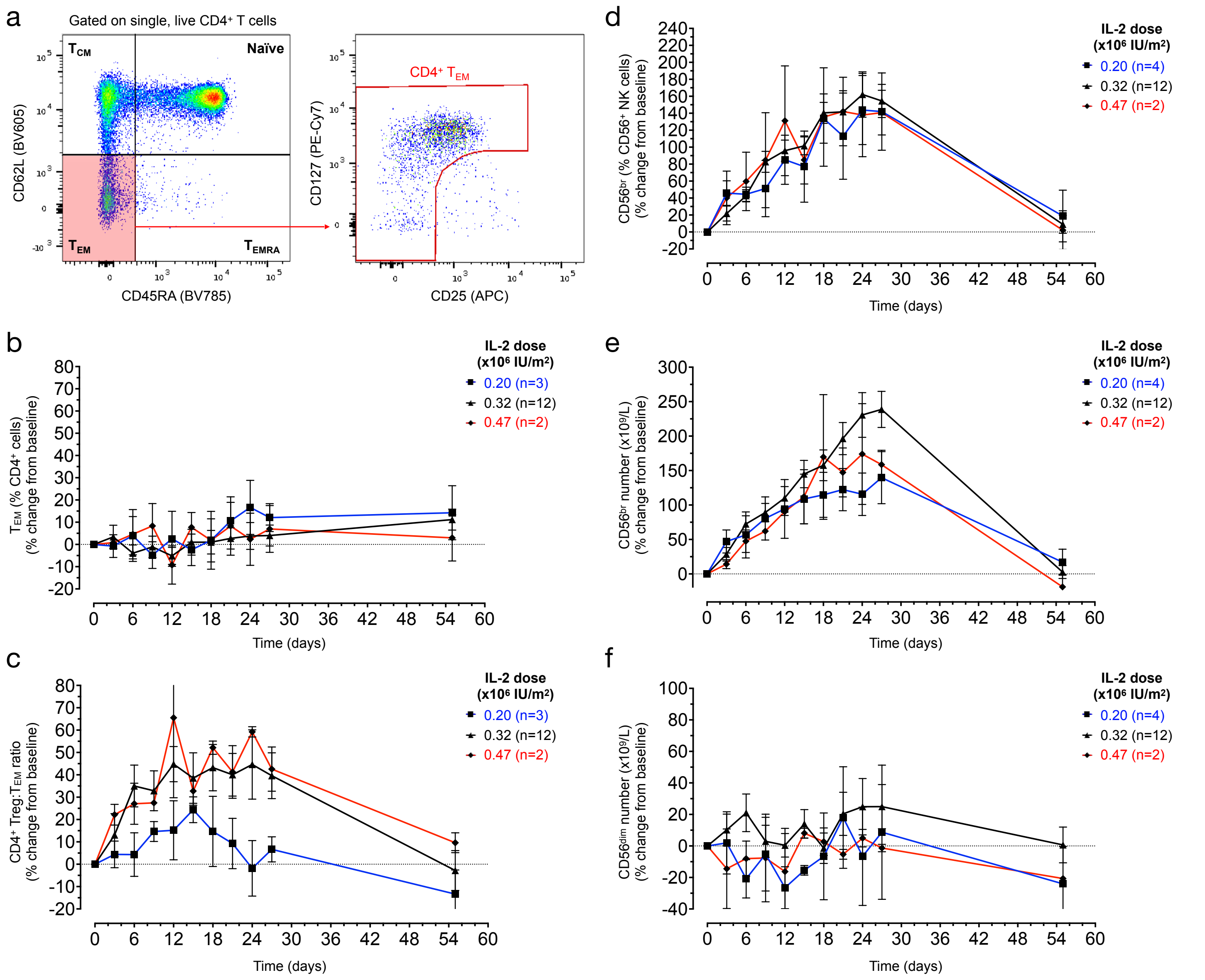

Supplementary Figure 1. Flow cytometric characterisation of the effector memory CD4$^+$ T and CD56$^+$ NK cell populations.

a, Gating strategy for the delineation of CD4$^+$ effector memory T cells (T_{EM}). **b-c**, Variation (depicted as the % change from pre-treatment baseline levels) in the frequency of CD4$^+$ T_{EM} (**b**), or the ratio between CD4$^+$ Tregs and T_{EM} cells (**c**) during the study. Baseline levels (mean values +/- SEM): CD4$^+$ T_{EM} = 10.4% +/- 1.39; CD4$^+$ Treg:T_{EM} ratio = 0.76 +/- 0.07. **d-f,** Variation (depicted as the % change from pre-treatment baseline levels) in the relative frequency of CD56^{br} NK cells within the total CD3$^-$CD56$^+$ NK cell gate (**d**), and in the absolute number of CD56^{br} (**e**) and CD56^{dim} (**f**) NK cells in blood during the study. Baseline levels (mean values +/- SEM): CD56^{br} = 6.2% +/- 0.55; CD56^{br} number (×10^9/L) = 0.011 +/- 0.001; CD56^{dim} Number (×10^9/L) = 0.18 +/- 0.02. In **b-f**, frequencies of the assessed immune subsets were determined by flow cytometry in n = 18 DILfrequency participants treated with the 3-day interval dosing schedule. Data are presented as mean values +/- SEM of the assessed immune subsets at each visit and were stratified according to the IL-2 dose. Source data for **b-f** are provided as a Source Data file.

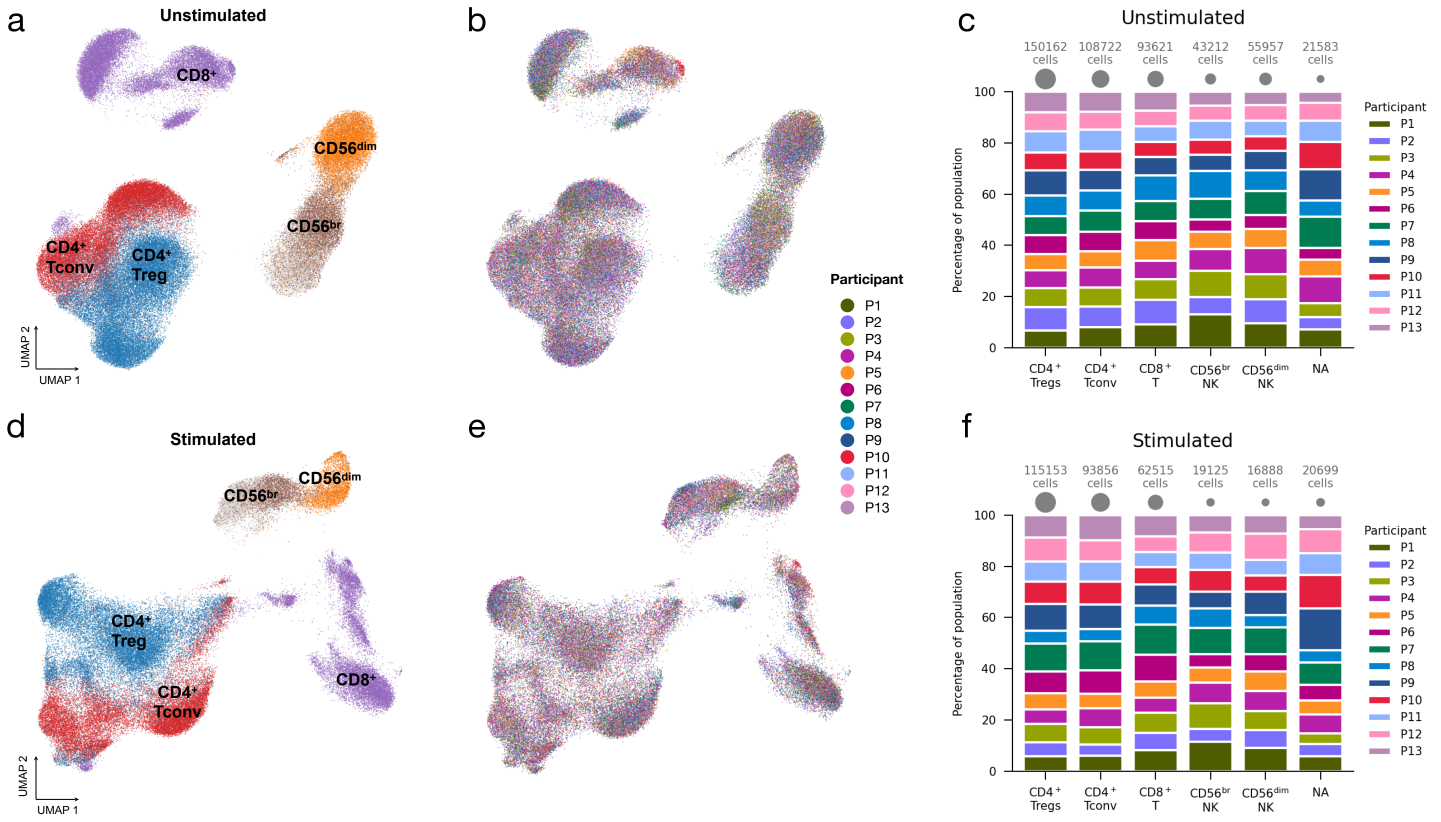

Supplementary Figure 2. Integration of the single-cell multiomics data.

a-b, Uniform Manifold Approximation and Projection (UMAP) plot depicting the clustering of the 460,941 unstimulated cells profiled in this study. Cells were coloured according to the corresponding oligo-conjugated barcode indicative of the respective FACS sorting gate (**a**), or according to the participant of origin (**b**). **c**, Relative proportion of cells from each individual participant in each of the flow-sorted unstimulated immune cell populations. Areas of grey circles represent total cell numbers in each immune cell population. **d-e**, UMAP plots depicting the clustering of the 323,839 cells profiled in this study following a short (90 min) in vitro stimulation with PMA + ionomycin. Cells were coloured according to the corresponding oligo-conjugated barcode indicative of the respective FACS sorting gate (**d**), or to the donor information (**e**). In **a** and **d**, gray dots correspond to untagged $CD56^{br}$ NK cells, whose sample barcode information are missing due to suboptimal sample tagging efficiency (see Methods). **f**, Relative proportion of cells from each individual participant in each of the flow-sorted stimulated immune cell populations. Areas of grey circles represent total cell numbers in each immune cell population. Source data for **c**, **f** are provided as a Source Data file.

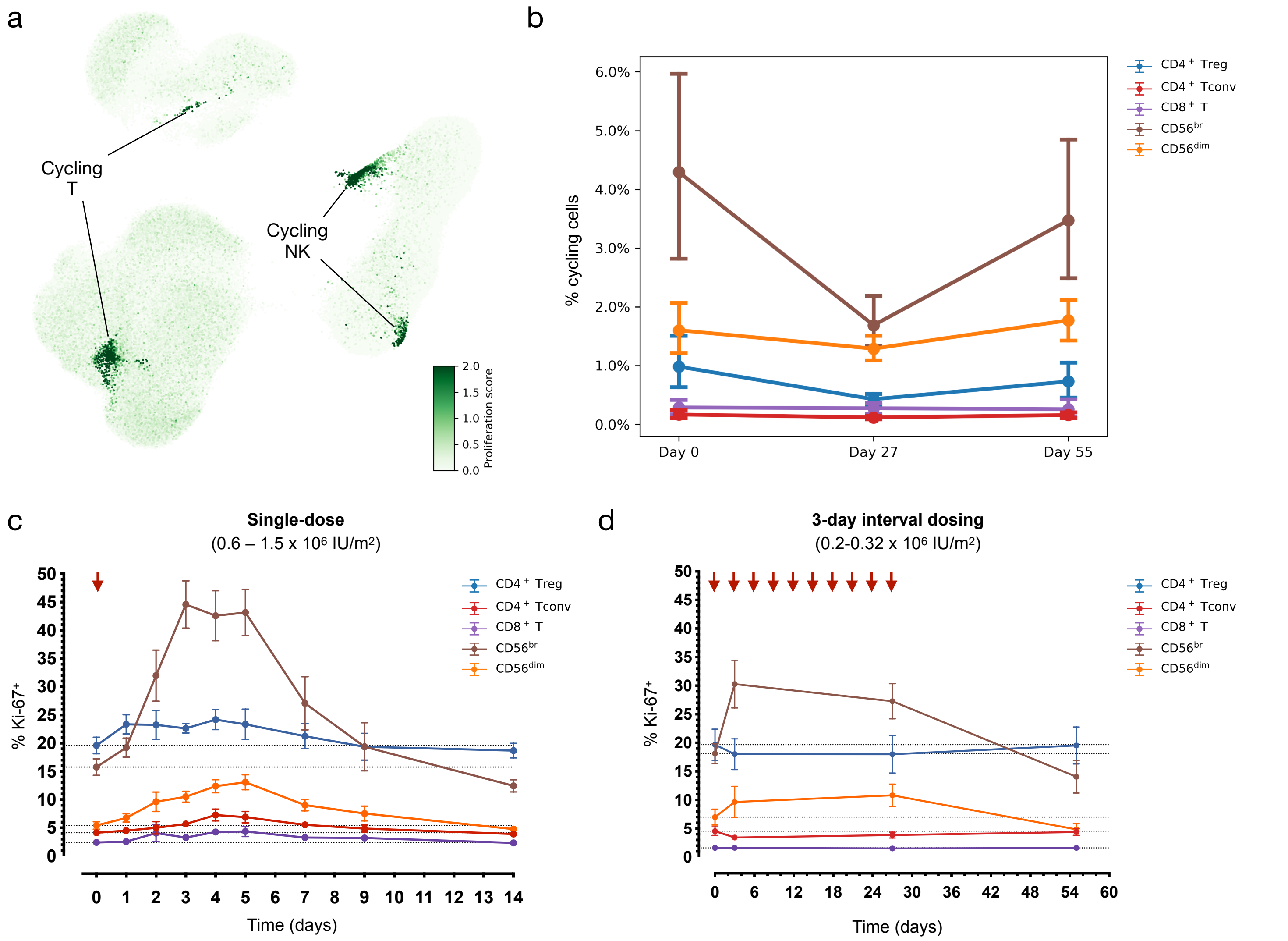

Supplementary Figure 3. Identification of proliferating cells.

a, UMAP plot depicting the expression of the cell-cycle signature score in each unstimulated cell. The cell-cycle signature score was calculated by summarising the expression levels of 11 pre-selected genes related to cell cycle. **b**, Frequency of proliferating cells in each immune subset identified by single-cell multiomics analysis. Proliferating cells were defined as the proportion of cells in the identified cycling clusters from each of the five sorted immune populations at Days 0, 27 and 55. Data are presented as mean values +/- 95% confidence interval. n = 13 participants. **c-d**, Frequency of Ki-67$^+$ cells within each of the five assessed immune populations in T1D patients treated with a single (DILT1D; **c**) or with the 3-day interval dosing schedule (DILfrequency; **d**). Ki-67 protein levels were assessed by intracellular flow cytometry in six patients from the DILT1D (treated with doses ranging from 0.6-1.5 x 10^6 IU/m^2 and profiled at 10 timepoints - Day 0, 1, 2, 3, 4, 5, 7, 9, 14 and 28) and five patients from the DILfrequency (treated with interval doses ranging from 0.2-0.32 x 10^6 IU/m^2 and profiled at four timepoints - Day 0, 3, 27 and 55). IL-2 dosing time points are depicted in dark red arrows. Data are presented as mean values +/- SEM. n = 13 participants. Source data for **b**, **c**, **d** are provided as a Source Data file.

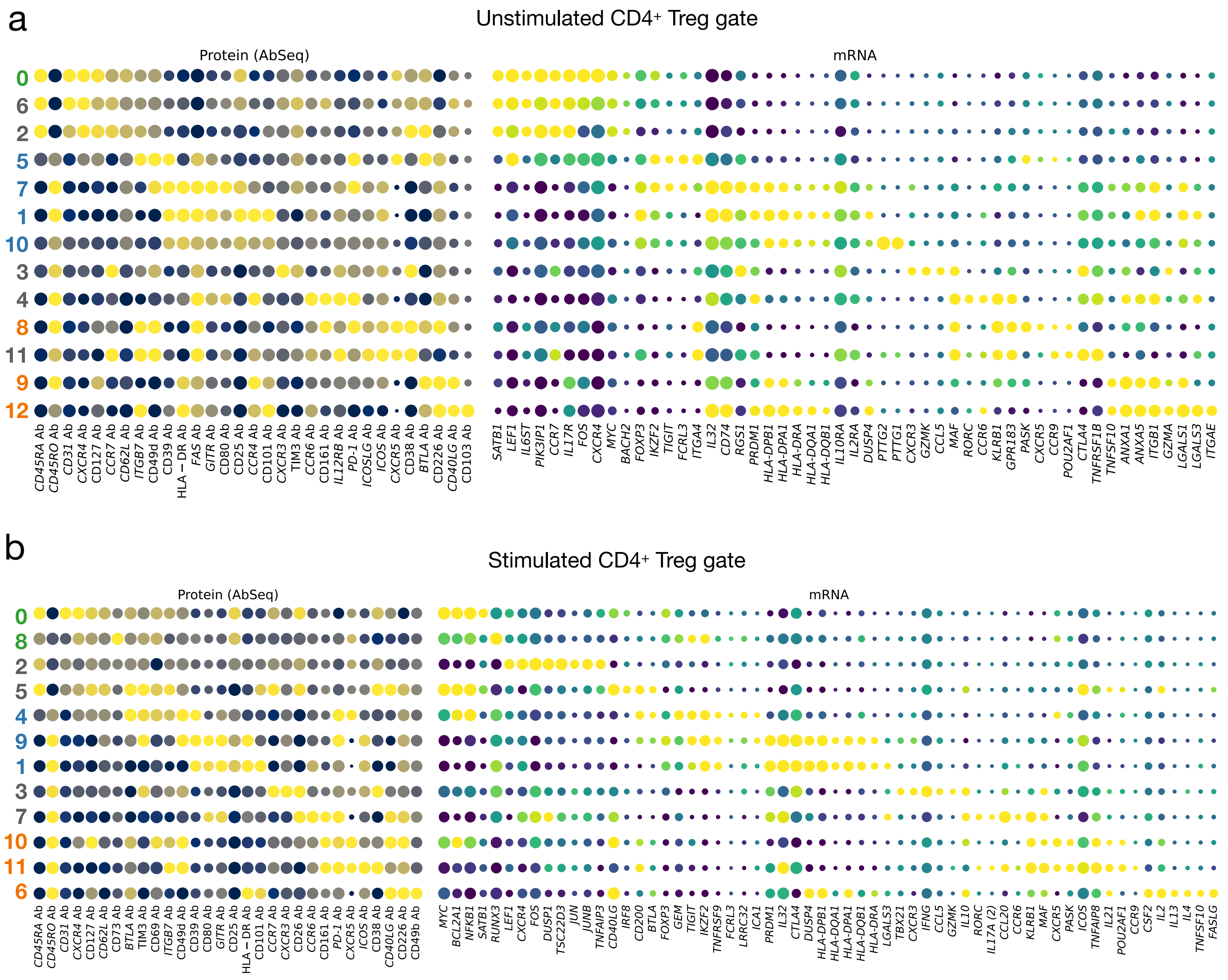

Supplementary Figure 4. Functional annotation of the $CD4^+$ $CD127^{low}CD25^{hi}$ T cell subsets.

a-b, Expression levels of selected differentially expressed markers delineating the functional subsets in the unstimulated (**a**) and in vitro stimulated (**b**) $CD4^+$ T cells sorted from the $CD127^{low}CD25^{hi}$ Treg gate. The size of the dots represents the frequency of the marker in the respective cluster and the colour brightness represents the relative expression level. Protein markers (AbSeq) are shown on the left section of the plots and mRNA markers on the right section. Clusters corresponding to: (i) naïve Tregs (defined as $CD45RA^+$ $FOXP3^+HELIOS^+$); (ii) memory Tregs (defined as $CD45RA^-$ $FOXP3^+HELIOS^+$); or (iii) $CD25^+$ $FOXP3^-HELIOS^-$ Teffs (defined as $CD45RA^-$ $FOXP3^-HELIOS^-$) are annotated in green, blue and orange, respectively. All other miscellaneous clusters not conforming to this definition are annotated in black.

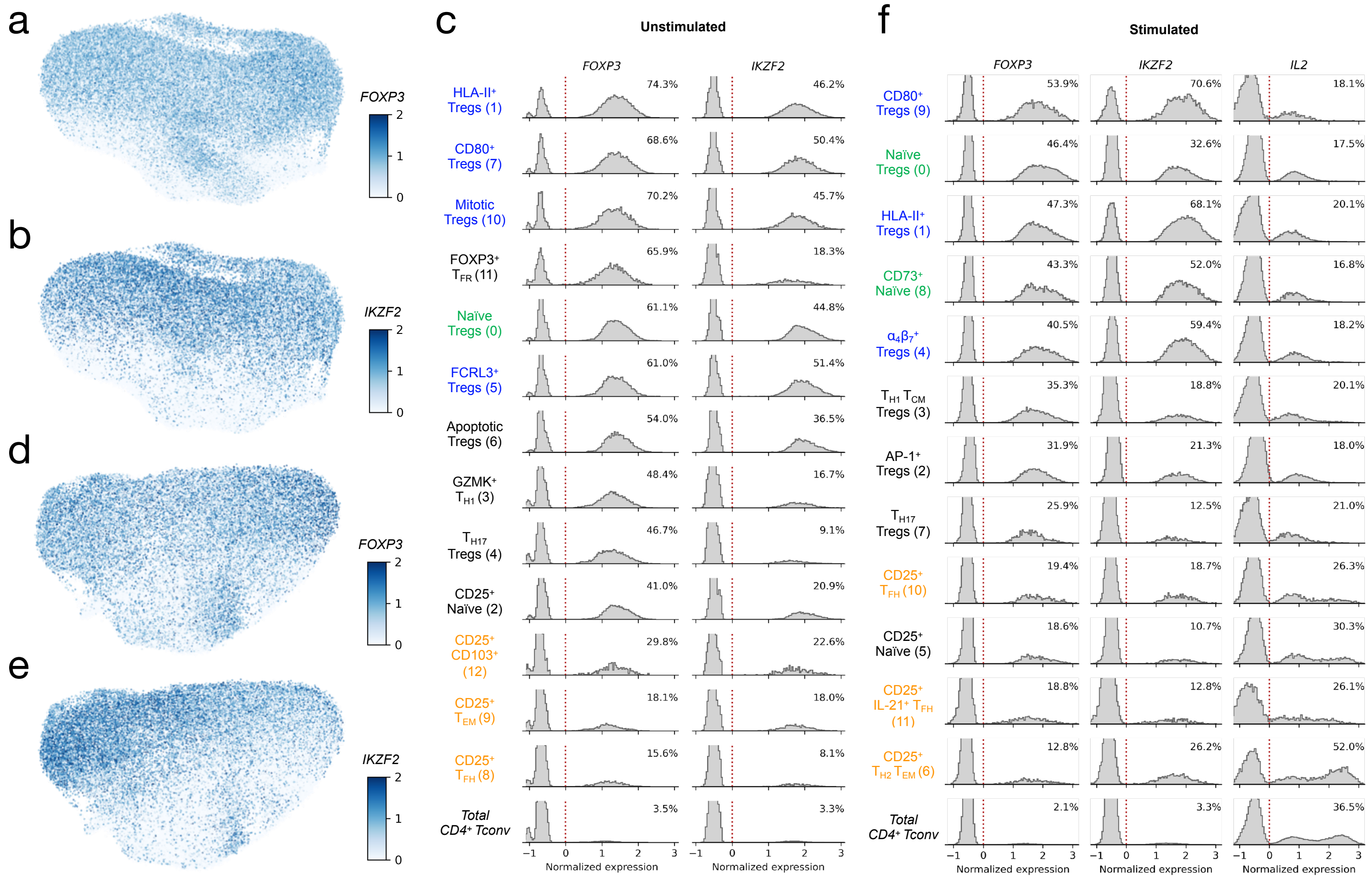

Supplementary Figure 5. Expression of *FOXP3*, *IKZF2* (HELIOS) and *IL2* differentiate functional CD4$^+$ Treg and CD4$^+$ Tconv subsets.

a-b, UMAP plots depicting the expression of the canonical Treg transcription factors *FOXP3* (**a**) and *IKZF2* (encoding HELIOS; **b**) in the unstimulated cells sorted from the CD4$^+$ CD127^{low}CD25^{hi} Treg gate. **c**, Distribution of expression levels of *FOXP3* and *IKZF2* in the identified unstimulated CD127^{low}CD25^{hi} CD4$^+$ T cell clusters. **d-e**, UMAP plots depicting the expression of the canonical Treg transcription factors *FOXP3* (**d**) and *IKZF2* (**e**) in the in vitro stimulated cells. **f**, Histograms depicting the expression of *FOXP3*, *IKZF2* and *IL2* in the identified in vitro stimulated clusters. Clusters corresponding to: (i) naïve Tregs (defined as CD45RA$^+$ FOXP3$^+$HELIOS$^+$); (ii) memory Tregs (defined as CD45RA$^-$ FOXP3$^+$HELIOS$^+$); or (iii) CD25$^+$ FOXP3$^-$HELIOS$^-$ Teffs (defined as CD45RA$^-$ FOXP3$^-$HELIOS$^-$) are annotated in green, blue and orange, respectively. All other miscellaneous clusters not conforming to this definition are annotated in black.

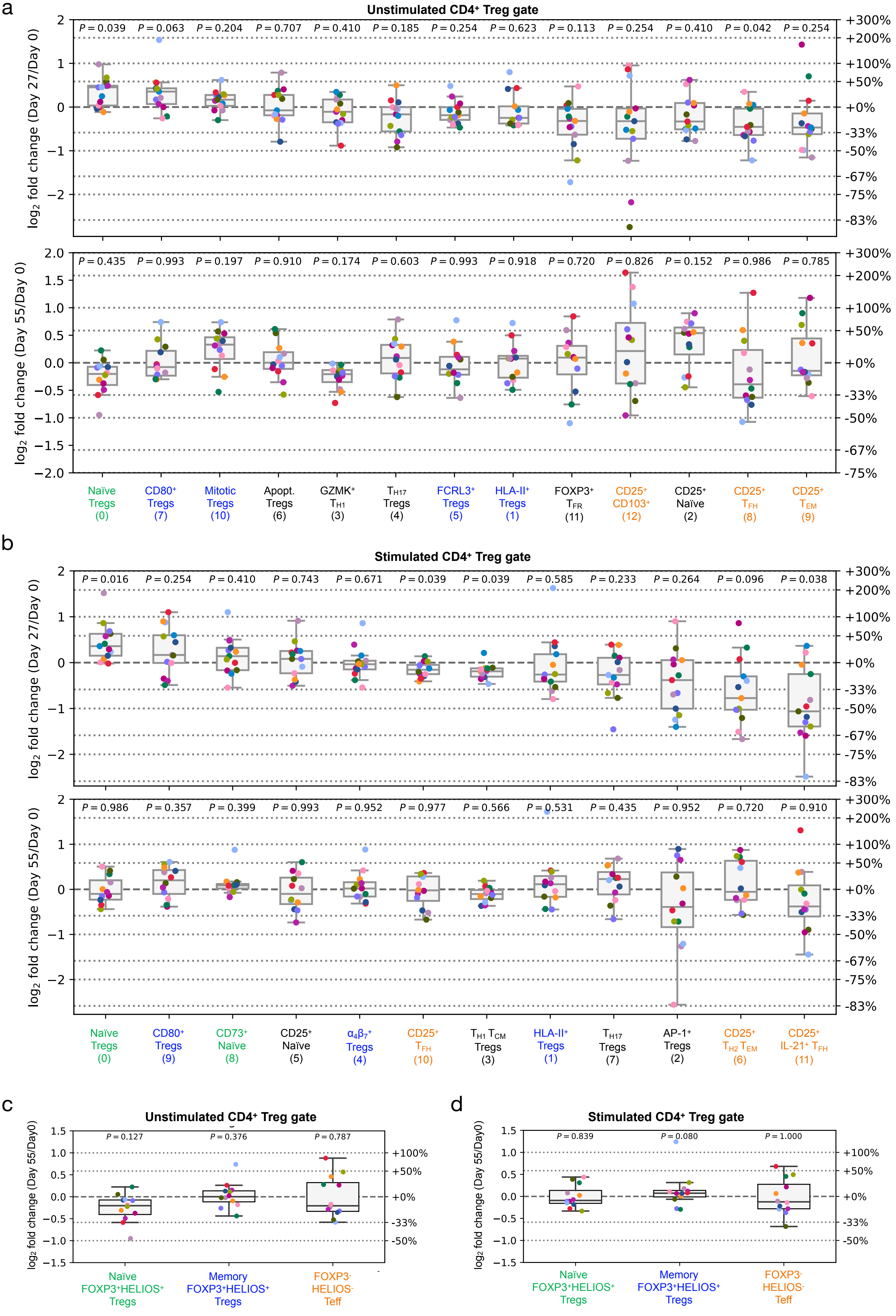

a
Unstimulated CD4+ Treg gate
P = 0.039 P = 0.063 P = 0.204 P = 0.707 P = 0.410 P = 0.185 P = 0.254 P = 0.623 P = 0.113 P = 0.254 P = 0.410 P = 0.042 P = 0.254
log2 fold change (Day 27/Day 0)
+300% +200% +100% +50% +0% -33% -50% -67% -75% -83%
P = 0.435 P = 0.993 P = 0.197 P = 0.910 P = 0.174 P = 0.603 P = 0.993 P = 0.918 P = 0.720 P = 0.826 P = 0.152 P = 0.986 P = 0.785
log2 fold change (Day 55/Day 0)
+300% +200% +100% +50% +0% -33% -50% -67% -75%
Naïve Tregs (0)
CD80+ Tregs (7)
Mitotic Tregs (10)
Apopt. Tregs (6)
GZMK+ TH1 (3)
TH17 Tregs (4)
FCRL3+ Tregs (5)
HLA-II+ Tregs (1)
FOXP3+ TFR (11)
CD25+ CD103+ (12)
CD25+ Naïve (2)
CD25+ TFH (8)
CD25+ TEM (9)
b
Stimulated CD4+ Treg gate
P = 0.016 P = 0.254 P = 0.410 P = 0.743 P = 0.671 P = 0.039 P = 0.039 P = 0.585 P = 0.233 P = 0.264 P = 0.096 P = 0.038
log2 fold change (Day 27/Day 0)
+300% +200% +100% +50% +0% -33% -50% -67% -75% -83%
P = 0.986 P = 0.357 P = 0.399 P = 0.993 P = 0.952 P = 0.977 P = 0.566 P = 0.531 P = 0.435 P = 0.952 P = 0.720 P = 0.910
log2 fold change (Day 55/Day 0)
+300% +200% +100% +50% +0% -33% -50% -67% -75% -83%
Naïve Tregs (0)
CD80+ Tregs (9)
CD73+ Naïve (8)
CD25+ Naïve (5)
α4β7+ Tregs (4)
CD25+ TFH (10)
TH1 TCM Tregs (3)
HLA-II+ Tregs (1)
TH17 Tregs (7)
AP-1+ Tregs (2)
CD25+ TH2 TEM (6)
CD25+ IL-21+ TFH (11)
c
Unstimulated CD4+ Treg gate
P = 0.127 P = 0.376 P = 0.787
log2 fold change (Day 55/Day0)
+100% +50% +0% -33% -50%
Naïve FOXP3+HELIOS+ Tregs
Memory FOXP3+HELIOS+ Tregs
FOXP3- HELIOS- Teff
d
Stimulated CD4+ Treg gate
P = 0.839 P = 0.080 P = 1.000
log2 fold change (Day 55/Day0)
+100% +50% +0% -33% -50%
Naïve FOXP3+HELIOS+ Tregs
Memory FOXP3+HELIOS+ Tregs
FOXP3- HELIOS- Teff

Supplementary Figure 6. Differential abundance analysis reveals a transient increased frequency of $FOXP3^+HELIOS^+$ Tregs following ld-IL2 treatment.

a-b, Relative frequency changes of all the identified unstimulated (**a**) or in vitro stimulated (**b**) $CD127^{low}CD25^{hi}$ T cell clusters between Day 0 and Day 27 (top panels) or Day 55 (bottom panels). Clusters corresponding to: (i) naïve Tregs (defined as $CD45RA^+$ $FOXP3^+HELIOS^+$), (ii) memory Tregs (defined as $CD45RA^-$ $FOXP3^+HELIOS^+$), and (iii) $CD25^+$ $FOXP3^-$ $HELIOS^-$ Teffs (defined as $CD45RA^-$ $FOXP3^-HELIOS^-$) are annotated in green, blue and orange, respectively. All other miscellaneous clusters not conforming to this definition are annotated in black. Asterisks denote statistically significant differences (two-sided Wilcoxon signed-rank test) in cell population frequencies between the two time points. **c-d**, Relative frequency changes of unstimulated (**c**) or in vitro stimulated (**d**) naïve $FOXP3^+HELIOS^+$ Treg, memory $FOXP3^+HELIOS^+$ Treg, and $CD25^+$ $FOXP3^-HELIOS^-$Teff clusters in blood between Day 0 and Day 55. In **a-d**, each dot represents cells from a single participant, with colour defined as in Supplementary Figure 2b. n = 13 participants. Each box ranges from the first quartile (Q1) to the third quartile (Q3), with a central line indicating the median. The bottom and top whiskers extend to the most extreme data points within Q1 − 1.5 × (Q3 − Q1) and Q3 + 1.5 × (Q3 − Q1), respectively. *P* values were calculated using two-sided Wilcoxon signed-rank tests based on cell population frequencies between the two assessed time points, followed by Benjamini–Hochberg FDR adjustment. Source data for **a-d** are provided as a Source Data file.

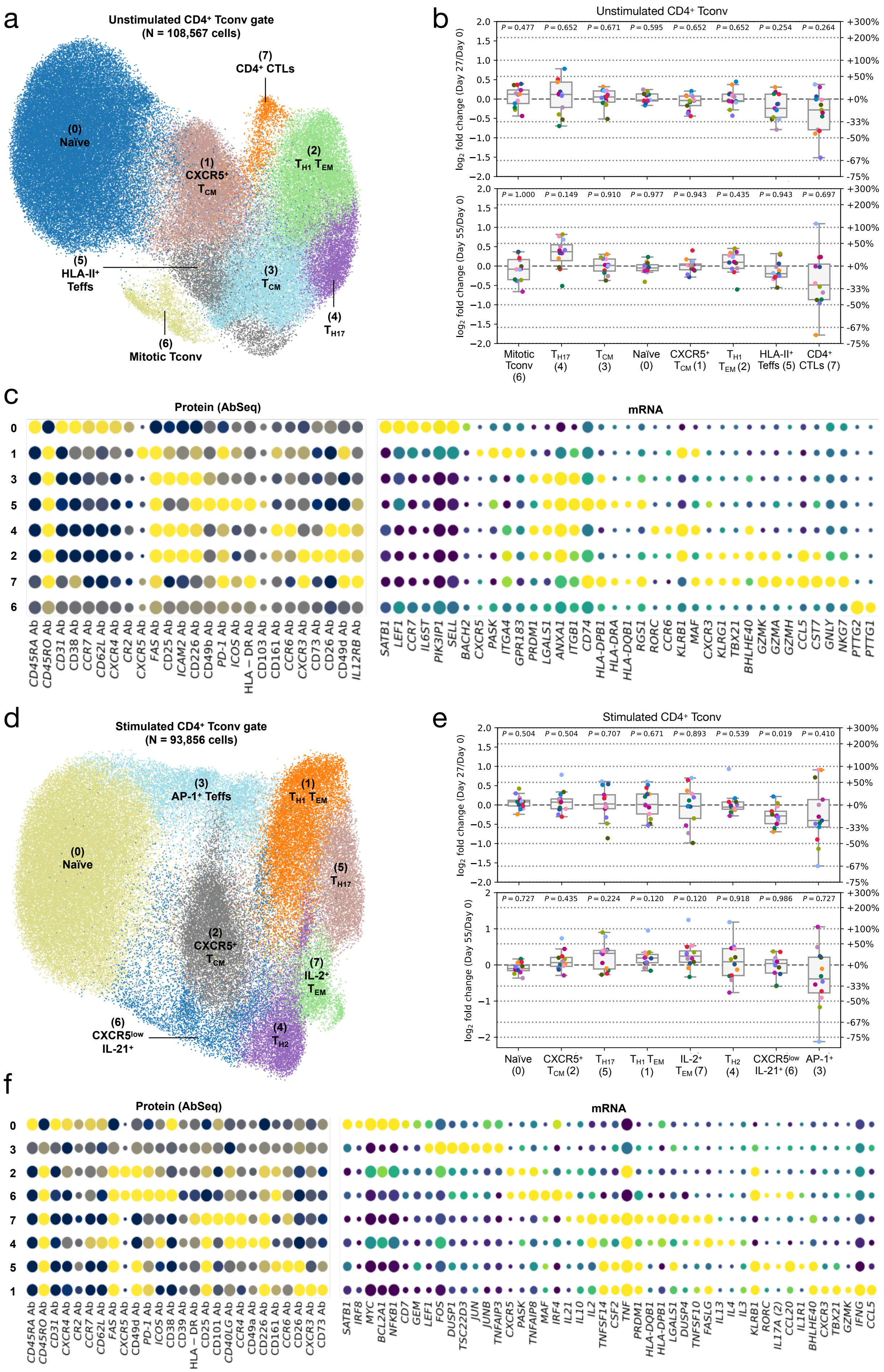
a
Unstimulated CD4+ Tconv gate
(N = 108,567 cells)
(0) Naïve
(1) CXCR5+ TCM
(2) TH1 TEM
(3) TCM
(4) TH17
(5) HLA-II+ Teffs
(6) Mitotic Tconv
(7) CD4+ CTLs
b
Unstimulated CD4+ Tconv
log2 fold change (Day 27/Day 0)
log2 fold change (Day 55/Day 0)
P = 0.477 P = 0.652 P = 0.671 P = 0.595 P = 0.652 P = 0.652 P = 0.254 P = 0.264
P = 1.000 P = 0.149 P = 0.910 P = 0.977 P = 0.943 P = 0.435 P = 0.943 P = 0.697
+300% +200% +100% +50% +0% -33% -50% -67% -75%
Mitotic Tconv (6)
TH17 (4)
TCM (3)
Naïve (0)
CXCR5+ TCM (1)
TH1 TEM (2)
HLA-II+ Teffs (5)
CD4+ CTLs (7)
c
Protein (AbSeq)
mRNA
CD45RA Ab, CD45RO Ab, CD31 Ab, CD38 Ab, CCR7 Ab, CD62L Ab, CXCR4 Ab, CR2 Ab, CXCR5 Ab, FAS Ab, CD25 Ab, ICAM2 Ab, CD226 Ab, CD49b Ab, PD-1 Ab, ICOS Ab, HLA – DR Ab, CD103 Ab, CD161 Ab, CCR6 Ab, CXCR3 Ab, CD73 Ab, CD26 Ab, CD49d Ab, IL12RB Ab
SATB1, LEF1, CCR7, IL6ST, PIK3IP1, SELL, BACH2, CXCR5, PASK, ITGA4, GPR183, PRDM1, LGALS1, ANXA1, ITGB1, CD74, HLA-DPB1, HLA-DRA, HLA-DQB1, RGS1, RORC, CCR6, KLRB1, MAF, CXCR3, KLRG1, TBX21, BHLHE40, GZMK, GZMA, GZMH, CCL5, CST7, GNLY, NKG7, PTTG2, PTTG1
d
Stimulated CD4+ Tconv gate
(N = 93,856 cells)
(0) Naïve
(1) TH1 TEM
(2) CXCR5+ TCM
(3) AP-1+ Teffs
(4) TH2
(5) TH17
(6) CXCR5low IL-21+
(7) IL-2+ TEM
e
Stimulated CD4+ Tconv
log2 fold change (Day 27/Day 0)
log2 fold change (Day 55/Day 0)
P = 0.504 P = 0.504 P = 0.707 P = 0.671 P = 0.893 P = 0.539 P = 0.019 P = 0.410
P = 0.727 P = 0.435 P = 0.224 P = 0.120 P = 0.120 P = 0.918 P = 0.986 P = 0.727
Naïve (0)
CXCR5+ TCM (2)
TH17 (5)
TH1 TEM (1)
IL-2+ TEM (7)
TH2 (4)
CXCR5low IL-21+ (6)
AP-1+ (3)
f
Protein (AbSeq)
mRNA
CD45RA Ab, CD45RO Ab, CD31 Ab, CXCR4 Ab, CR2 Ab, CCR7 Ab, CD62L Ab, FAS Ab, CXCR5 Ab, CD49d Ab, PD-1 Ab, ICOS Ab, CD38 Ab, CD39 Ab, HLA – DR Ab, CD25 Ab, CD101 Ab, CD40LG Ab, CCR4 Ab, CD49a Ab, CD226 Ab, CD161 Ab, CCR6 Ab, CD26 Ab, CXCR3 Ab, CD73 Ab
SATB1, IRF8, MYC, BCL2A1, NFKB1, CD7, GEM, LEF1, FOS, DUSP1, TSC22D3, JUN, JUNB, TNFAIP3, CXCR5, PASK, TNFAIP8, MAF, IRF4, IL21, IL10, IL2, TNFSF14, CSF2, TNF, PRDM1, HLA-DQB1, HLA-DPB1, LGALS1, DUSP4, TNFSF10, FASLG, IL13, IL4, IL3, KLRB1, RORC, IL17A (2), CCL20, IL1R1, BHLHE40, CXCR3, TBX21, GZMK, IFNG, CCL5

Supplementary Figure 7. Analysis of circulating $CD4^+$ $CD25^{–/low}$ conventional T cells.

a, UMAP plot depicting the clustering of the 108,722 unstimulated $CD4^+$ T cells sorted from the $CD25^{–/low}$ Tconv gate profiled in this study. Clusters were manually annotated based on the expression of key differentially expressed mRNA and protein markers. **b**, Expression levels of selected differentially expressed markers delineating the functional subsets in the unstimulated $CD4^+$ T cells sorted from the $CD25^{–/low}$ Tconv gate. The size of the dots represents the frequency of the marker in the respective cluster and the colour brightness represents the relative expression level. Protein markers (AbSeq) are shown on the left section of the plots and mRNA markers on the right section. **c,** Relative frequency changes of all identified unstimulated $CD4^+$ $CD25^{–/low}$ Tconv clusters in blood at Day 27 (top panel) or Day 55 (bottom panel) compared to the baseline pre-treatment (Day 0) levels. **d-f**, Plots corresponding to **a-c**, for the 93,856 in vitro stimulated $CD4^+$ T cells sorted from the $CD25^{–/low}$ Tconv gate. In **b** and **e**, each box ranges from the first quartile (Q1) to the third quartile (Q3), with a central line indicating the median. The bottom and top whiskers extend to the most extreme data points within Q1 − 1.5 × (Q3 − Q1) and Q3 + 1.5 × (Q3 − Q1), respectively. Each participant is represented by a different colour, as defined in Supplementary Figure 2b. n = 13 participants. *P* values were calculated using two-sided Wilcoxon signed-rank tests based on cell population frequencies between the two assessed time points, followed by Benjamini-Hochberg FDR adjustment. Source data for **b**, **e** are provided as a Source Data file.

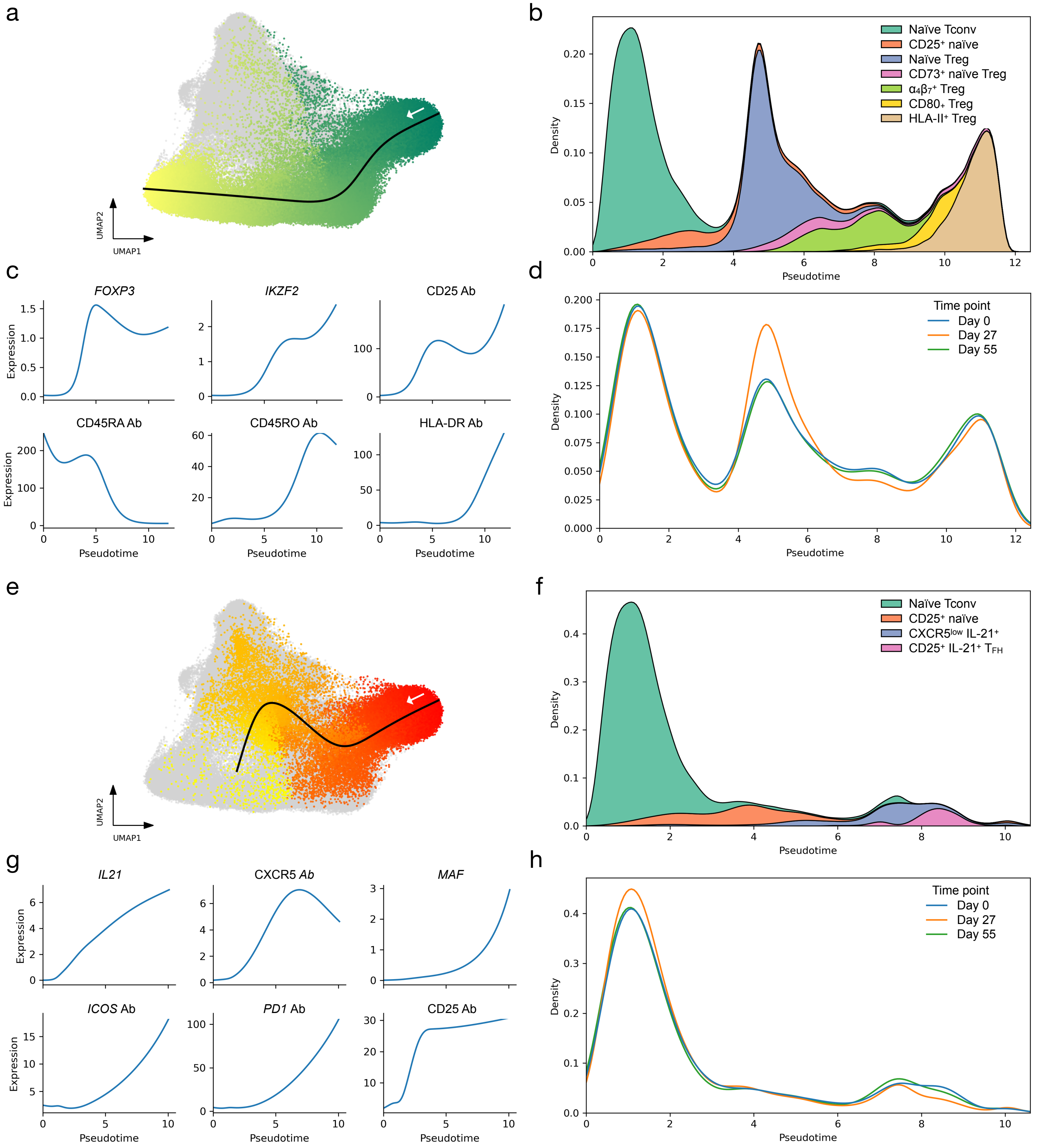

Supplementary Figure 8. Pseudo-time analysis reveals trajectories of Treg and T_{FH} differentiation in in vitro stimulated $CD4^+$ T cells.

a, UMAP plot depicting the Treg differentiation trajectory identified by Slingshot pseudo-time analysis. **b**, Pseudo-time distribution of cells associated with the Treg trajectory, stratified by cluster labels. **c**, Fitted expression curves of selected

canonical Treg genes by pseudo-time. **d**, Pseudo-time distribution of cells associated with the Treg trajectory, stratified by time points. **e**, UMAP plot depicting the T_{FH} differentiation trajectory identified by Slingshot pseudo-time analysis. **f**, Pseudo-time distribution of cells associated with the T_{FH} trajectory, stratified by cluster labels. **g**, Fitted expression curves of selected canonical T_{FH} genes by pseudo-time. **h**, Pseudo-time distribution of cells associated with the T_{FH} trajectory, stratified by time points. Data from **a-h** were generated from in vitro stimulated $CD4^+$ T cells.

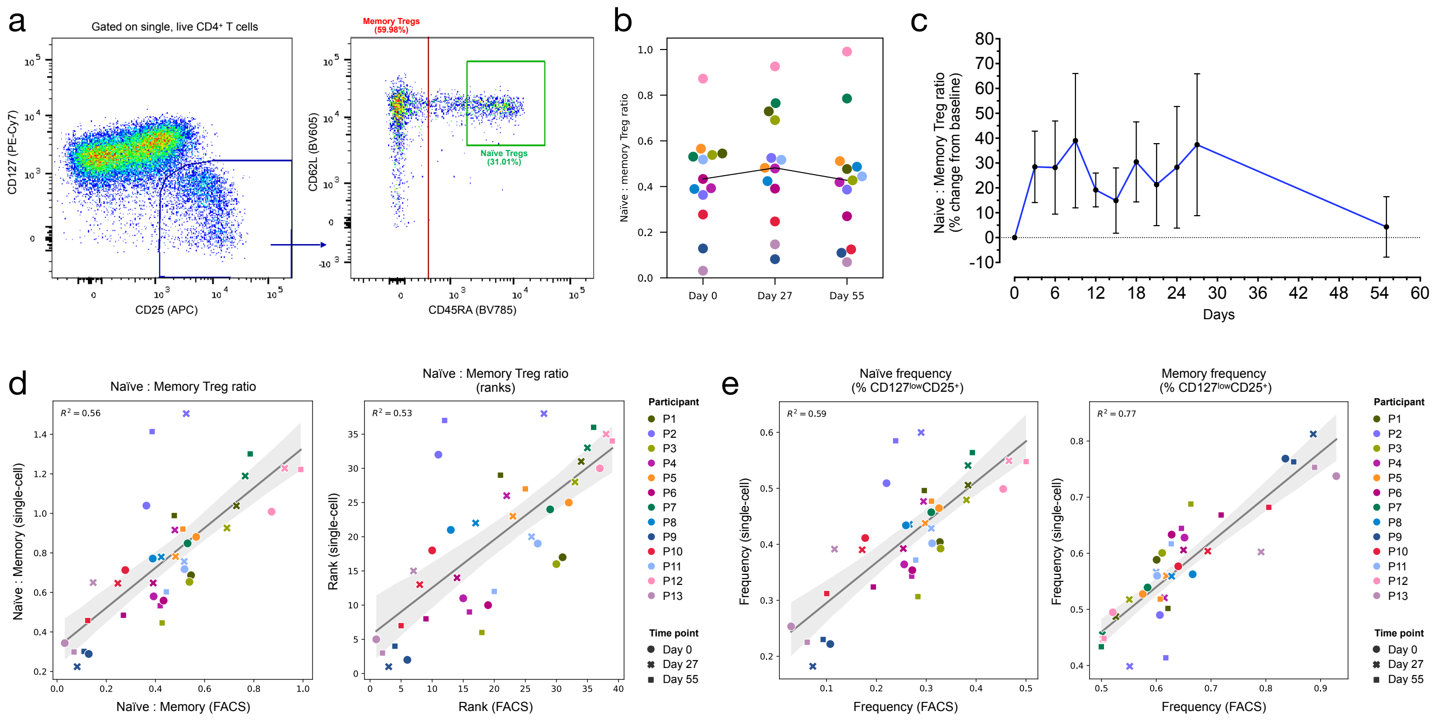

Supplementary Figure 9. Replication of the ratio of naïve:memory Tregs by FACS.

a, Gating strategy for the delineation of the naïve (CD45RA$^+$) and memory (CD45RA$^-$) compartment by flow cytometry within cells sorted from the CD4$^+$ CD127^{low}CD25^{hi} Treg gate. **b**, Ratio between naïve and memory cells within the CD4$^+$ CD127^{low}CD25^{hi} Treg gate at the three assessed timepoints (Day 0, Day 27 and Day 55). Each dot represents cells from a single participant. The connecting line represents the median naïve:memory ratio in the 13 participants selected for single-cell analysis at each time point. **c**, Variation (depicted as the % change from baseline pre-treatment levels) in the naïve:memory ratio within the CD4$^+$ CD127^{low}CD25^{hi} Treg gate assessed by flow cytometry. Data are presented as mean values +/- SEM at each visit for n = 13 participants selected for single-cell analysis. **d**, Correlation between the naïve:memory ratio within CD4$^+$ CD127^{low}CD25^{hi} T cells (left panel), or the corresponding ranks (right panel), obtained from FACS data (x axis) and that estimated from single-cell sequencing data (y axis). **e**, Correlation between the frequency of naïve (CD45RA$^+$; left panel) and memory (CD45RA$^-$; right panel) cells within CD127^{low}CD25^{hi} T cells obtained from FACS data (x axis) and that obtained from single-cell sequencing data (y axis). In **b**, **d** and **e**, each dot represents a sample, with colours and shapes representing participants and time points of origin, respectively. In **d-e**, linear regression models are presented as least square estimates (black lines) +/- 95% confidence intervals (grey shades). n = 13 participants. Source data for **b-e** are provided as a Source Data file.

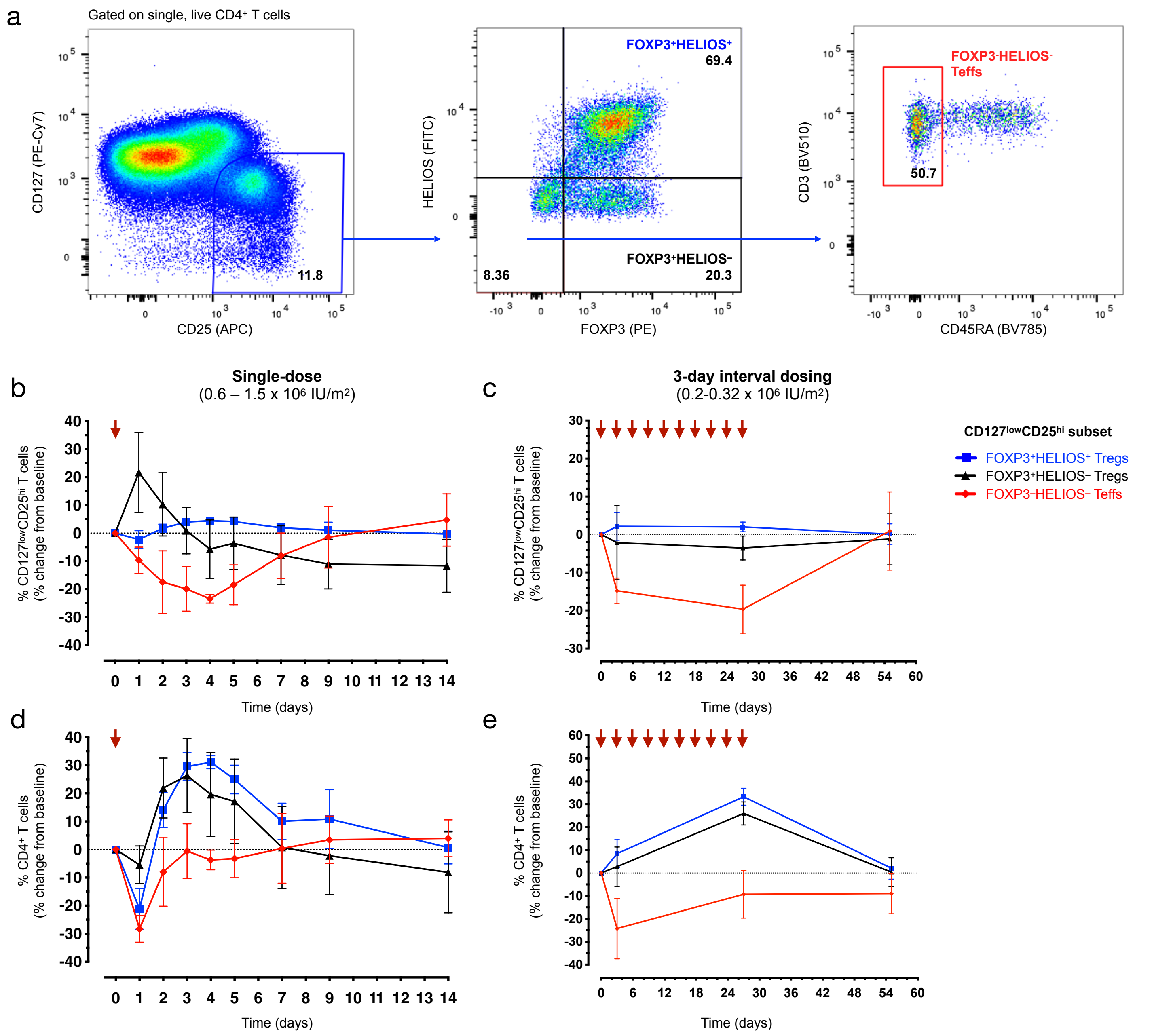

Supplementary Figure 10. Delineation of Treg subsets by FACS.

a, Gating strategy for the delineation of the FOXP3$^+$HELIOS$^+$ Treg, FOXP3$^+$HELIOS$^-$ Treg and CD45RA$^-$ FOXP3$^-$HELIOS$^-$ Teff subsets by flow cytometry. **b**-**c** Variation (depicted as the % change from baseline pre-treatment levels) in the frequency of FOXP3$^+$HELIOS$^+$ (blue), FOXP3$^+$HELIOS$^-$ (black) and CD45RA$^-$ FOXP3$^-$HELIOS$^-$ (red) cells within the CD127^{low}CD25^{hi} gate in patients treated with a single dose (DILT1D; **b**) or with the 3-day interval dosing schedule (DILfrequency; **c**). Baseline levels (mean values +/- SEM): (i) DILT1D: FOXP3$^+$HELIOS$^+$ = 71.5% +/- 1.47, FOXP3$^+$HELIOS$^-$ = 15.6% +/- 1.63 and CD45RA$^-$ FOXP3$^-$HELIOS$^-$ = 4.7% +/- 0.44; (ii) DILfrequency: FOXP3$^+$HELIOS$^+$ = 66.9% +/- 2.0, FOXP3$^+$HELIOS$^-$ = 19.8% +/- 1.60 and CD45RA$^-$ FOXP3$^-$HELIOS$^-$ = 8.0% +/- 1.02. **d-e**, Variation (depicted as the % change from baseline pre-treatment levels) in the frequency of the FOXP3$^+$HELIOS$^+$ Treg, FOXP3$^+$HELIOS$^-$ Treg and CD45RA$^-$ FOXP3$^-$HELIOS$^-$ Teff subsets in patients treated with a single dose (**d**) or with the 3-day interval (e) dosing schedule. Baseline levels (mean values +/- SEM): (i)

DILT1D: $FOXP3^+HELIOS^+$ = 5.0% +/- 0.54, $FOXP3^+HELIOS^-$ = 1.2% +/- 0.24 and $CD45RA^-$ $FOXP3^-HELIOS^-$ = 0.34% +/- 0.06; (ii) DILfrequency: $FOXP3^+HELIOS^+$ = 4.2% +/- 0.44, $FOXP3^+HELIOS^-$ = 1.3% +/- 0.22 and $CD45RA^-$ $FOXP3^-HELIOS^-$ = 0.49% +/- 0.05. In **b** and **d**, n = 6 DILT1D participants treated with doses ranging from 0.6-1.5 x 106 IU/m2. In **c** and **e**, n = 5 DILfrequency participants treated with interval doses ranging from 0.2-0.32 x 106 IU/m2. In **b**-**e**, data are presented as mean values +/- SEM. IL-2 dosing time points are depicted in dark red arrows. Source data for **b-e** are provided as a Source Data file.

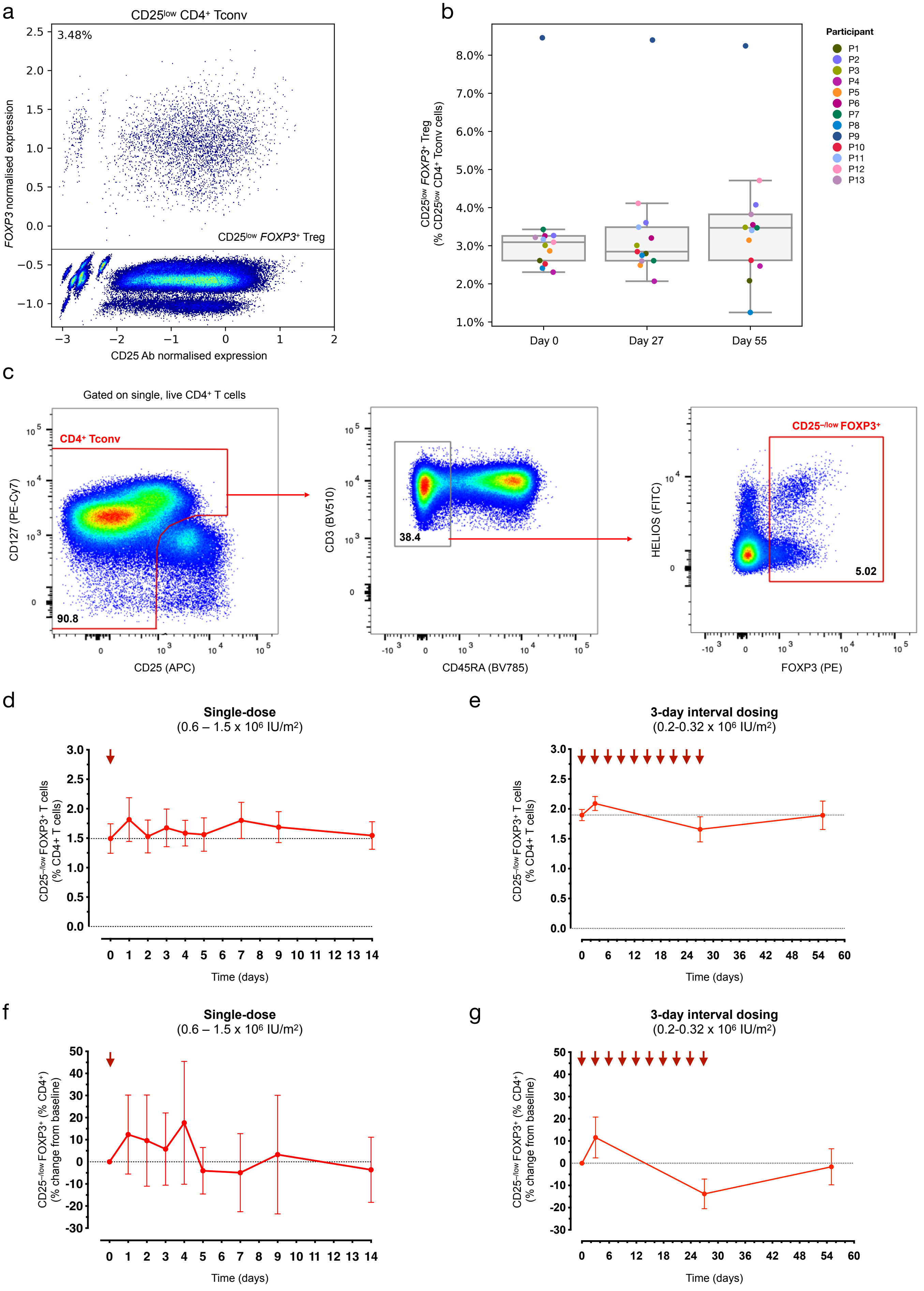

a
CD25low CD4+ Tconv
3.48%
FOXP3 normalised expression
CD25low FOXP3+ Treg
CD25 Ab normalised expression
b
CD25low FOXP3+ Treg (% CD25low CD4+ Tconv cells)
Day 0
Day 27
Day 55
Participant
P1
P2
P3
P4
P5
P6
P7
P8
P9
P10
P11
P12
P13
c
Gated on single, live CD4+ T cells
CD4+ Tconv
90.8
CD127 (PE-Cy7)
CD25 (APC)
38.4
CD3 (BV510)
CD45RA (BV785)
CD25−/low FOXP3+
5.02
HELIOS (FITC)
FOXP3 (PE)
d
Single-dose
(0.6 – 1.5 x 10^6 IU/m^2)
CD25−/low FOXP3+ T cells (% CD4+ T cells)
Time (days)
e
3-day interval dosing
(0.2-0.32 x 10^6 IU/m^2)
CD25−/low FOXP3+ T cells (% CD4+ T cells)
Time (days)
f
Single-dose
(0.6 – 1.5 x 10^6 IU/m^2)
CD25−/low FOXP3+ (% CD4+) (% change from baseline)
Time (days)
g
3-day interval dosing
(0.2-0.32 x 10^6 IU/m^2)
CD25−/low FOXP3+ (% CD4+) (% change from baseline)
Time (days)

Supplementary Figure 11. Interval low-dose IL-2 treatment does not affect the frequency of CD25$^{-/low}$ FOXP3^{+} T cells.

a, Identification of FOXP3^{+} within cells sorted from the CD25$^{-/low}$ Tconv gate. FOXP3^{+} cells were defined as cells with one or more counts of *FOXP3* RNA detected. Data was generated from the analysis of the 13 DILfrequency donors selected for the single-cell multiomics analysis. **b**, Box and whiskers plot depicts the frequency of FOXP3^{+} cells within the CD25$^{-/low}$ Tconv gate at Days 0, 27 and 55. Each dot represents a sample, with colours and shapes representing participants and time points of origin, respectively. Each box ranges from the first quartile (Q1) to the third quartile (Q3), with a central line indicating the median. The bottom and top whiskers extend to the most extreme data points within Q1 − 1.5 × (Q3 − Q1) and Q3 + 1.5 × (Q3 − Q1), respectively. n = 13 participants. **c**, Gating strategy for the delineation of CD25$^{-/low}$ FOXP3^{+} T cells by flow cytometry. **d**-**e** Frequency of CD25$^{-/low}$ FOXP3^{+} T cells in patients treated with a single dose (DILT1D; **d**) or with the 3-day interval dosing schedule (DILfrequency; **e**). **f-g**, Variation (depicted as the % change from baseline pre-treatment levels) in the frequency of the CD25$^{-/low}$ FOXP3^{+}T cells in patients treated with a single dose (**f**) or with the 3-day interval dosing (g) schedule. In **d** and **f**, n = 6 DILT1D participants treated with doses ranging from 0.6-1.5 x 106 IU/m2. In **e** and **g**, n = 5 DILfrequency participants treated with interval doses ranging from 0.2-0.32 x 106 IU/m2. In **d-g**, data are presented as mean values +/- SEM. IL-2 dosing days are depicted in red in the x axis. Source data for **b**, **d-g** are provided as a Source Data file.

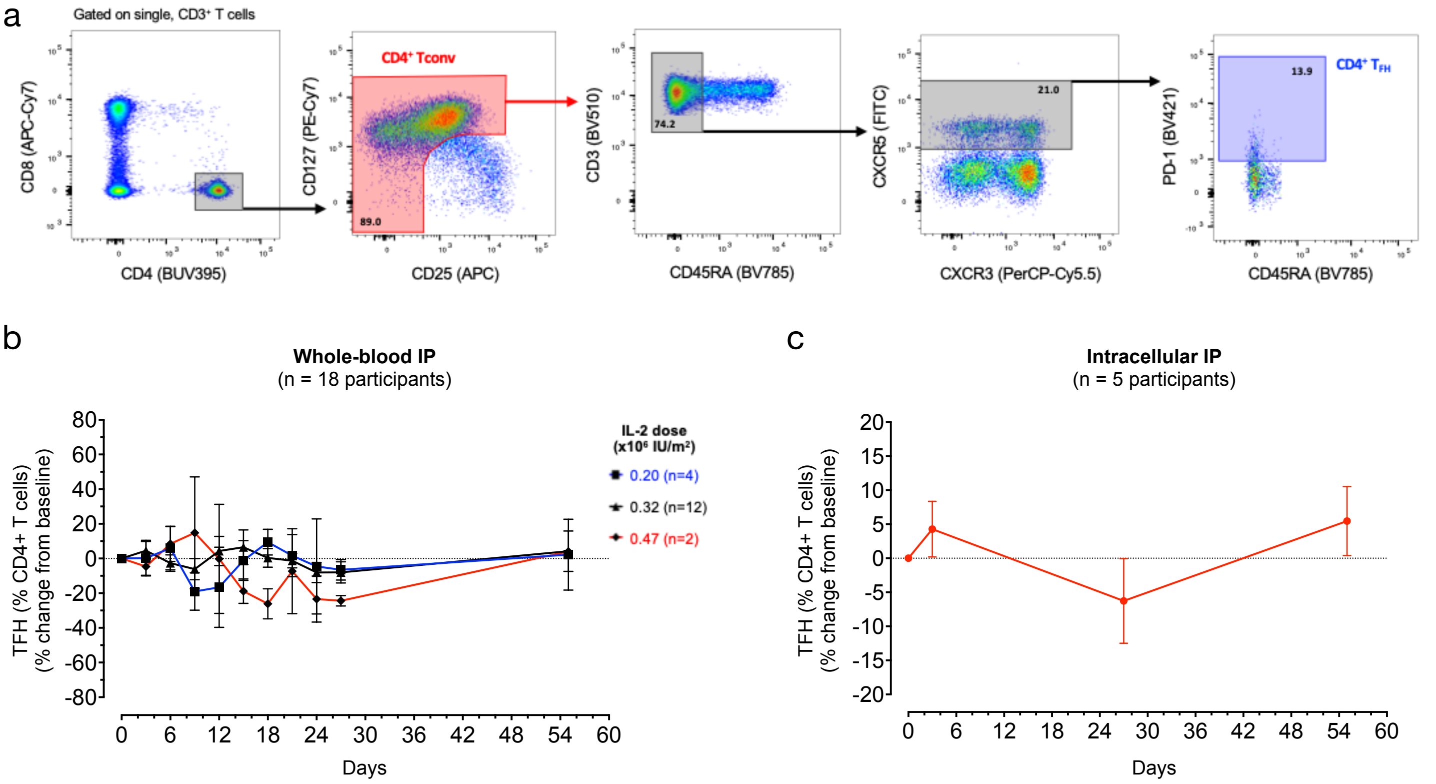

Supplementary Figure 12. Immunophenotyping the circulating CD4$^+$ T follicular helper (T_{FH}) subset by FACS.

a, Gating strategy for the delineation of circulating CD4$^+$ T_{FH} cells by FACS. **b-c**, Variation (depicted as the % change from pre-treatment baseline levels) in the relative frequency of CD4$^+$ T_{FH} cells. Frequency of CD4$^+$ T_{FH} cells was determined by flow cytometry in whole-blood from n = 18 DILfrequency participants treated with the 3-day interval dosing schedule (**b**) or in a subset of n = 5 DILfrequency participants selected for intracellular immunophenotyping (IP, **c**) who were treated with 200,000-320,000 IU/m^2 IL-2 every three days. Data are presented mean values +/- SEM.

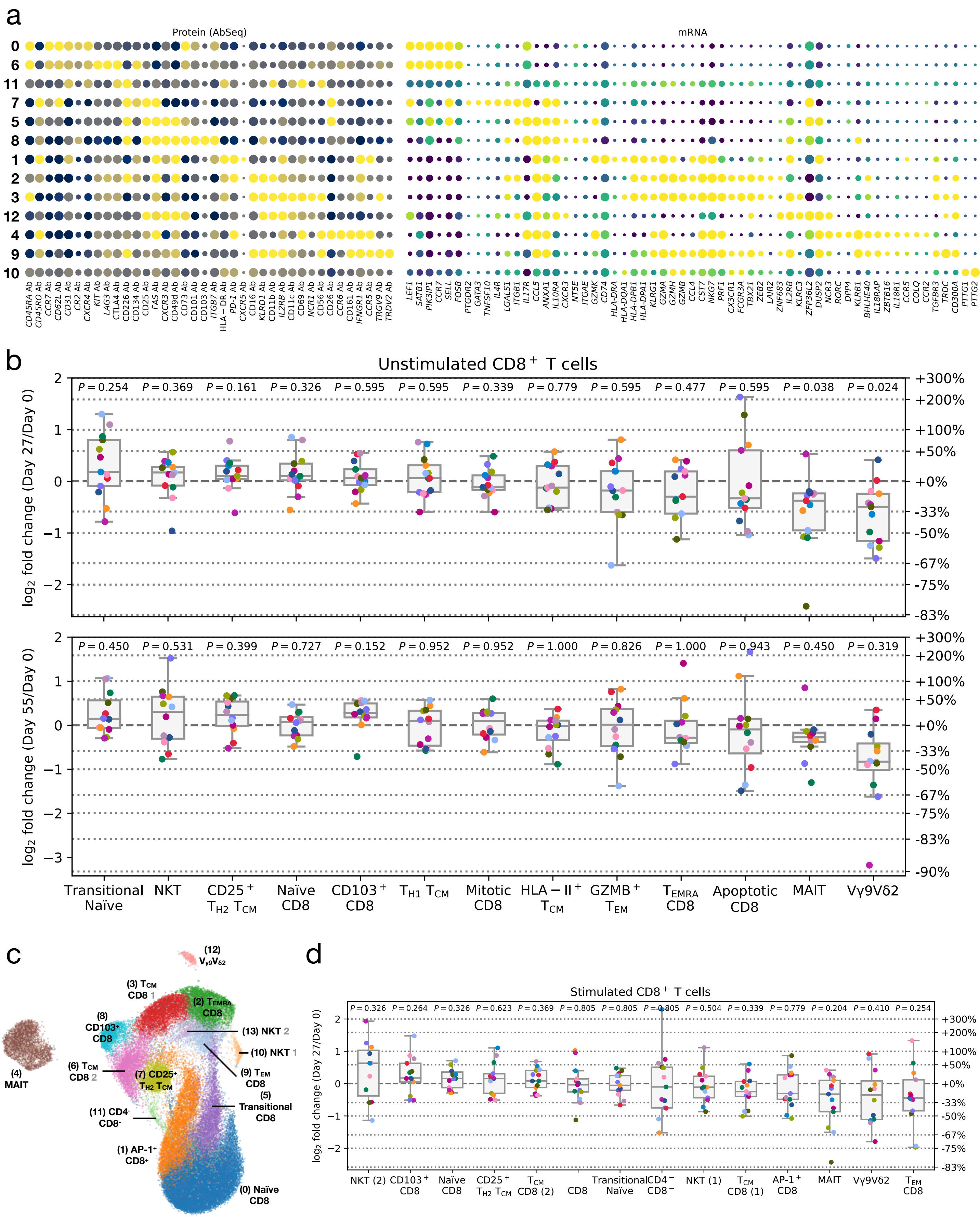
a
Protein (AbSeq)
mRNA
0
6
11
7
5
8
1
2
3
12
4
9
10
CD45RA Ab
CD45RO Ab
CCR7 Ab
CD62L Ab
CD31 Ab
CR2 Ab
CXCR4 Ab
KIT Ab
LAG3 Ab
CTLA4 Ab
CD226 Ab
CD134 Ab
CD25 Ab
FAS Ab
CXCR3 Ab
CD49d Ab
CD73 Ab
CD101 Ab
CD103 Ab
ITGB7 Ab
HLA − DR Ab
PD-1 Ab
CXCR5 Ab
CD16 Ab
KLRD1 Ab
CD11b Ab
IL2RB Ab
CD11c Ab
CD69 Ab
NCR1 Ab
CD56 Ab
CD26 Ab
CCR6 Ab
CD161 Ab
IFNGR1 Ab
CCR5 Ab
TRGV9 Ab
TRDV2 Ab
LEF1
SATB1
PIK3IP1
CCR7
SELL
FOSB
PTGDR2
GATA3
TNFSF10
IL4R
LGALS1
ITGB1
IL17R
CCL5
ANXA1
IL10RA
CXCR3
NT5E
ITGAE
GZMK
CD74
HLA-DRA
HLA-DQA1
HLA-DPB1
HLA-DPA1
KLRG1
GZMA
GZMH
GZMB
CCL4
CST7
NKG7
PRF1
CX3CR1
FCGR3A
TBX21
ZEB2
LAIR2
ZNF683
IL2RB
KLRC3
ZFP36L2
DUSP2
NCR3
RORC
DPP4
KLRB1
BHLHE40
IL18RAP
ZBTB16
IL18R1
CCR5
COLQ
CCR2
TGFBR3
TRDC
CD300A
PTTG1
PTTG2
b
Unstimulated CD8 + T cells
P = 0.254
P = 0.369
P = 0.161
P = 0.326
P = 0.595
P = 0.595
P = 0.339
P = 0.779
P = 0.595
P = 0.477
P = 0.595
P = 0.038
P = 0.024
log2 fold change (Day 27/Day 0)
+300%
+200%
+100%
+50%
+0%
-33%
-50%
-67%
-75%
-83%
P = 0.450
P = 0.531
P = 0.399
P = 0.727
P = 0.152
P = 0.952
P = 0.952
P = 1.000
P = 0.826
P = 1.000
P = 0.943
P = 0.450
P = 0.319
log2 fold change (Day 55/Day 0)
-90%
Transitional Naïve
NKT
CD25 + TH2 TCM
Naïve CD8
CD103 + CD8
TH1 TCM
Mitotic CD8
HLA − II + TCM
GZMB + TEM
TEMRA CD8
Apoptotic CD8
MAIT
Vγ9Vδ2
c
(12) Vγ9Vδ2
(3) TCM CD8 1
(2) TEMRA CD8
(8) CD103+ CD8
(13) NKT 2
(10) NKT 1
(4) MAIT
(6) TCM CD8 2
(7) CD25+ TH2 TCM
(9) TEM CD8
(5) Transitional CD8
(11) CD4- CD8-
(1) AP-1+ CD8+
(0) Naïve CD8
d
Stimulated CD8 + T cells
P = 0.326
P = 0.264
P = 0.326
P = 0.623
P = 0.369
P = 0.805
P = 0.805
P = 0.805
P = 0.504
P = 0.339
P = 0.779
P = 0.204
P = 0.410
P = 0.254
log2 fold change (Day 27/Day 0)
+300%
+200%
+100%
+50%
+0%
-33%
-50%
-67%
-75%
-83%
NKT (2)
CD103 + CD8
Naïve CD8
CD25 + TH2 TCM
TCM CD8 (2)
CD8
Transitional Naïve
CD4 − CD8 −
NKT (1)
TCM CD8 (1)
AP-1 + CD8
MAIT
Vγ9Vδ2
TEM CD8

Supplementary Figure 13. Functional annotation of $CD8^+$ T cells.

a, Expression of selected differentially-expressed markers delineating the functional subsets identified from the 13 clustering of the 93,621 unstimulated $CD8^+$ T cells profiled in this study. The size of the dots represents the frequency of the marker in the respective cluster and the colour brightness represents the relative expression level. Protein markers (AbSeq) are shown on the left section of the plots and mRNA markers on the right section. **b,** Relative frequency changes of all identified $CD8^+$ T cell clusters on Day 27 (top panel) or Day 55 (bottom panel) compared to the baseline pre-treatment levels. **c**, UMAP plot depicting the clustering of in vitro stimulated $CD8^+$ T cells. Clusters were manually annotated based on the expression of key differentially expressed mRNA and protein markers. **d**, Relative frequency changes of the 14 identified stimulated $CD8^+$ T cell clusters at Day 27 compared to the baseline pre-treatment (Day 0) levels. In **b** and **d**, each box ranges from the first quartile (Q1) to the third quartile (Q3), with a central line indicating the median. The bottom and top whiskers extend to the most extreme data points within $Q1 - 1.5 \times (Q3 - Q1)$ and $Q3 + 1.5 \times (Q3 - Q1)$, respectively. Each participant is represented by a different colour, as defined in Supplementary Figure 2b. n = 13 participants. Source data for **b**, **d** are provided as a Source Data file.

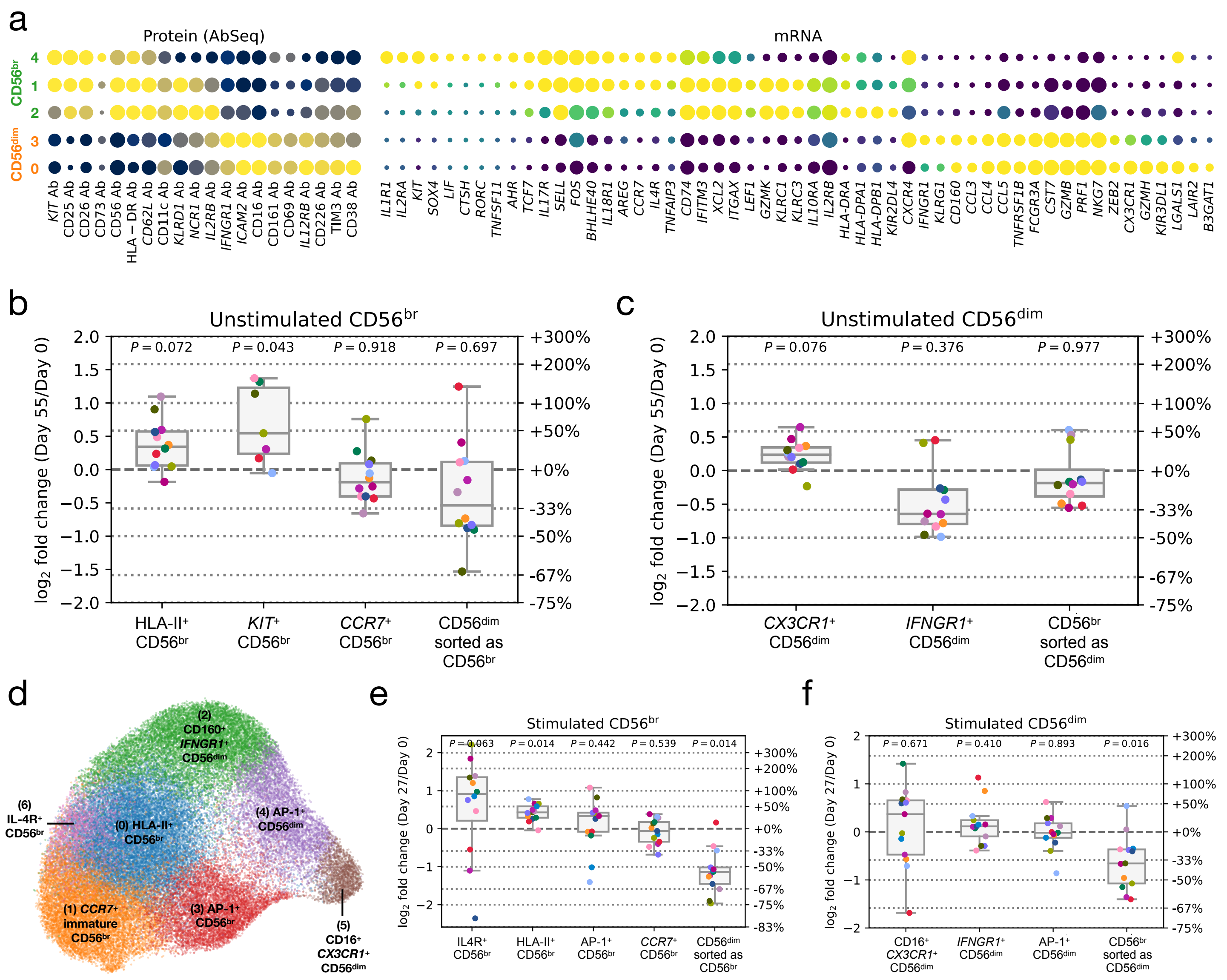

Supplementary Figure 14. Functional annotation of CD56⁺ NK cells.

a, Expression of selected differentially-expressed markers delineating the five functional subsets identified from the clustering of the 99,169 unstimulated CD56⁺ NK cells profiled in this study. The size of the dots represents the frequency of the marker in the respective cluster and the colour brightness represents the relative expression level. Protein markers (AbSeq) are shown on the left section of the plots and mRNA markers on the right section. The dotted red line separates the clusters corresponding to either CD56^{br} (top) and CD56^{dim} NK cells (bottom). **b-c**, Relative frequency changes of all identified unstimulated CD56^{br} (**b**) and CD56^{dim} (**c**) NK cell clusters on Day 55 compared to the baseline pre-treatment levels. **d**, UMAP plot depicts the clustering of in vitro stimulated CD56⁺ NK cells. **e-f** Relative frequency changes of all identified clusters of cells sorted from either the CD56^{br} (**e**) or CD56^{dim} (**f**) NK cell gates following in vitro stimulation on Day 27 compared to the baseline pre-treatment (Day 0) levels. In **b**, **c**, **e**, and **f**, each box ranges from the first quartile (Q1) to the third quartile (Q3), with a central line indicating the median. The bottom and top whiskers extend to the most extreme data points within Q1 − 1.5 × (Q3 − Q1) and Q3 + 1.5 × (Q3 − Q1), respectively. Each participant is represented by

a different colour, as defined in Supplementary Figure 2b. n = 13 participants. Source data for **b**, **c**, **e**, **f** are provided as a Source Data file.

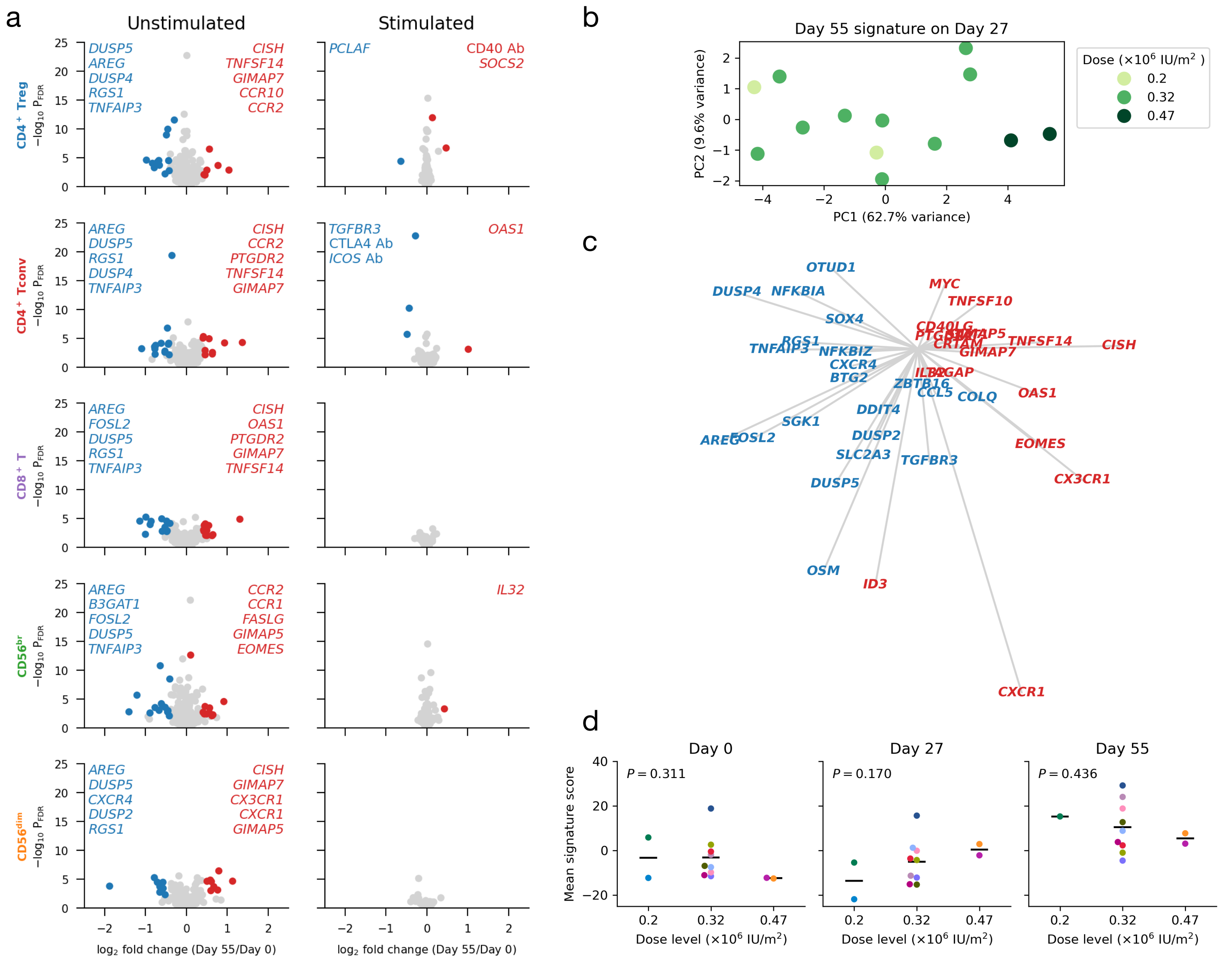

Supplementary Figure 15. Induction of the anti-inflammatory gene expression signature at Day 55 is IL-2 dose-dependent and is detected in all T and NK cell populations.

a, Volcano plots depicting gene expression changes between Day 0 and Day 55 for the five assessed immune populations, for unstimulated (left panels) and stimulated (right panels) cells. Significantly differentially expressed genes are coloured in red (upregulated genes) or blue (downregulated genes). The top five up- and downregulated genes (as defined by fold change) are labelled on each panel. Fold change and P values were calculated using a generalized linear model implemented in DESeq2 (see Methods). **b**, Principal component analysis (PCA) correlating gene expression changes with dose levels. PCA was performed on $\log_2$ fold-change values of Day 55 signature genes on Day 27 in $CD8^+$ T cells. Each dot represents a participant, with colours indicating IL-2 dose levels. Vectors representing the gene loading scores of PC1 and PC2 are shown on the right panel, with genes up- and downregulated on Day 55 shown in red and blue, respectively. **c**, Day 55 signature scores by participant for each time point. Each participant is represented by a different colour. **d**, Day 55 signature scores plotted against IL-2 dose. The average value of the Day 55 signature score in the five cell types is shown for each participant. Each participant is represented by a different colour, as defined in Supplementary Figure 2b. n = 13

participants. *P* values were calculated using linear regression, without multiple-comparison adjustments. Source data for **a-d** are provided as a Source Data file.
