# Supplementary material for: Low-dose IL-2 reduces IL-21+ T cell frequency and induces anti-inflammatory gene expression in type 1 diabetes
Source: Nat Commun. 2022 Nov 28;13:7324. doi: 10.1038/s41467-022-34162-3 (PMC9705541; doi:10.1038/s41467-022-34162-3)
Supplement: Supplementary file 3 — Description of Additional Supplementary Files [file 41467_2022_34162_MOESM3_ESM.pdf]

Description of Additional Supplementary Files

File Name: Supplementary Data 1
Description: Summary of the baseline characteristics of study participants.

File Name: Supplementary Data 2
Description: Complete list of antibodies used in this study and gene content of the scRNA-seq targeted mRNA panel.

File Name: Supplementary Data 3
Description: Summary of the cluster-specific RNA and AbSeq markers used for cell type annotation. Fold change and *P* values were calculated using a using a negative binomial generalized linear model implemented in Seurat (see Methods).

File Name: Supplementary Data 4
Description: Differential expression calculated on all annotated clusters between Day 0 and Day 27 or Day 55 analysed using DESeq2. Fold change and *P* values were calculated using a generalized linear model implemented in DESeq2 (see Methods).
