# Supplementary material for: Low-dose IL-2 reduces IL-21+ T cell frequency and induces anti-inflammatory gene expression in type 1 diabetes
Source: Nat Commun. 2022 Nov 28;13:7324. doi: 10.1038/s41467-022-34162-3 (PMC9705541; doi:10.1038/s41467-022-34162-3)
Supplement: Supplementary file 8 — Reporting Summary [file 41467_2022_34162_MOESM8_ESM.pdf]

nature portfolio

Corresponding author(s): Ricardo Ferreira & John Todd

Last updated by author(s): Oct 11, 2022

Reporting Summary

Nature Portfolio wishes to improve the reproducibility of the work that we publish. This form provides structure for consistency and transparency in reporting. For further information on Nature Portfolio policies, see our Editorial Policies and the Editorial Policy Checklist.

Statistics

For all statistical analyses, confirm that the following items are present in the figure legend, table legend, main text, or Methods section.

n/a	Confirmed	
☐	☒	The exact sample size (n) for each experimental group/condition, given as a discrete number and unit of measurement
☐	☒	A statement on whether measurements were taken from distinct samples or whether the same sample was measured repeatedly
☐	☒	The statistical test(s) used AND whether they are one- or two-sided<br>*Only common tests should be described solely by name; describe more complex techniques in the Methods section.*
☐	☒	A description of all covariates tested
☐	☒	A description of any assumptions or corrections, such as tests of normality and adjustment for multiple comparisons
☐	☒	A full description of the statistical parameters including central tendency (e.g. means) or other basic estimates (e.g. regression coefficient) AND variation (e.g. standard deviation) or associated estimates of uncertainty (e.g. confidence intervals)
☐	☒	For null hypothesis testing, the test statistic (e.g. F, t, r) with confidence intervals, effect sizes, degrees of freedom and P value noted<br>*Give P values as exact values whenever suitable.*
☒	☐	For Bayesian analysis, information on the choice of priors and Markov chain Monte Carlo settings
☒	☐	For hierarchical and complex designs, identification of the appropriate level for tests and full reporting of outcomes
☐	☒	Estimates of effect sizes (e.g. Cohen's d, Pearson's r), indicating how they were calculated

Our web collection on statistics for biologists contains articles on many of the points above.

Software and code

Policy information about availability of computer code

Data collection	Flow cytometry data was acquired on a BD LSR Fortessa instrument (BD Biosciences). Cell sorting for single-cell RNA-sequencing was performed on FACSAria Fusion sorter (BD Biosciences). Cell capture and library preparation for single-cell RNA-sequencing was performed using the BD Rhapsody single-cell system (BD Bioscience). Sequencing was performed on NovaSeq 6000 instrument (Illumina).
Data analysis	The following software were used in this study:<br>FlowJo v10.8.1<br>BD FACSDiva v8.0.1<br>GraphPad Prism v9.3.1<br>apeglm v1.12.0<br>DESeq2 v1.30.1<br>Seurat v4.0.0<br>Bowtie2 v2.4.4<br>Slingshot v1.8.0<br>tradeSeq v1.4.0<br>The custom code used in the study for data analyses and visualisation has been deposited in FigShare (https://doi.org/10.6084/m9.figshare.21395214).

For manuscripts utilizing custom algorithms or software that are central to the research but not yet described in published literature, software must be made available to editors and reviewers. We strongly encourage code deposition in a community repository (e.g. GitHub). See the Nature Portfolio guidelines for submitting code & software for further information.

Data

Policy information about availability of data

All manuscripts must include a data availability statement. This statement should provide the following information, where applicable:

- Accession codes, unique identifiers, or web links for publicly available datasets
- A description of any restrictions on data availability
- For clinical datasets or third party data, please ensure that the statement adheres to our policy

Single-cell sequencing data generated in this study are available from Gene Expression Omnibus (GEO; accession number GSE201197). Source Data for the main and supplementary Figures are provided in the manuscript.

Field-specific reporting

Please select the one below that is the best fit for your research. If you are not sure, read the appropriate sections before making your selection.

☒ Life sciences ☐ Behavioural & social sciences ☐ Ecological, evolutionary & environmental sciences

For a reference copy of the document with all sections, see nature.com/documents/nr-reporting-summary-flat.pdf

Life sciences study design

All studies must disclose on these points even when the disclosure is negative.

Sample size

Single-cell RNA-sequencing data were collected from 13 participants profiled at three timepoints each (Days 0, 27 and 55). Sample size was selected to maximise the number of patients with PBMC samples available treated with a 3-day interval with IL-2 doses ranging from 200,000 - 470,000 IU/m2.

Whole-blood flow cytometry data were collected from 18 participants. Sample size was selected to include all 18 study participants from the DILfrequency study (ClinicalTrials.gov Identifier: NCT02265809) included in the 3-day interval group and treated with IL-2 with doses ranging from 200,000 - 470,000 IU/m2.

Validation of the single-cell multiomics data was performed by flow cytometry in two follow up cohorts: (i) a single LD-IL-2 dosing cohort, consisting of 6 T1D patients treated with doses ranging from 600,000 - 1,500,000 IU/m2 from the DILT1D study (ClinicalTrials.gov Identifier: NCT01827735). Each patient was profiled at 10 timepoints (Day 0, 1, 2, 3, 4, 5, 7, 9, 14 and 28); and (ii) a multiple dosing cohort, consisting of 5 T1D patients enrolled from the DILfrequency study and treated with the 3-day dosing interval (dose range 200,000-320,000 IU/m2). Each patient was profiled at 4 timepoints (Day 0, 3, 27 and 55).

Data exclusions

The Day 55 visit from Participant 8 was excluded from the single-cell RNA-seq analysis. Due to sample quality reasons, this sample yielded virtually no cells for analysis, and was therefore removed from the analysis (as described in the Methods section). Additional standard scRNA-seq data quality control steps were performed, which are described in detail in the Methods section. No other data were excluded from the analysis.

Replication

Reproducibility of the single-cell multiomics data was ensured by maximising the number of biological replicates and by comparing longitudinal samples (day 0, day 27 and day 55 visits) from each participant.

Replication of selected cellular phenotypes identified through single-cell multiomics were replicated by flow cytometry in two follow up cohorts (see 'Sample size' section for details). Namely, the following cellular phenotypes were replicated by FACS analyses:

1) Proliferating (Ki-67+) cells among the CD4+ Treg, CD4+ Tconv, CD8+ T, CD56br NK and CD56dim NK cell subsets: in the single LD-IL-2 dosing replication cohort (DILT1D; Supplementary Fig. 3c - n = 6 patients profiled at 10 timepoints); and in the multiple dosing replications cohort (DILfrequency; Supplementary Fig. 3d - n = 5 patients profiled at 4 timepoints).

2) Naive : Memory Treg ratio: in the 13 DILfrequency participants selected for single-cell multiomics (Supplementary Fig. 9c). Correlation with single-cell multiomics data shown in Supplementary Fig. 9d,e).

3) CD4+ CD127lowCD25hi T cell subsets (FOXP3+HELIOS+; FOXP3+HELIOS- and FOXP3-HELIOS-): in the single LD-IL-2 dosing replication cohort (DILT1D; Supplementary Fig. 10b,d - n = 6 patients profiled at 10 timepoints); and in the multiple dosing replication cohort (DILfrequency; Supplementary Fig. 10c,e - n = 5 patients profiled at 4 timepoints).

4) CD25-/low FOXP3+ T cells: in the single LD-IL-2 dosing replication cohort (DILT1D; Supplementary Fig. 11d,f - n = 6 patients profiled at 10 timepoints); and in the multiple dosing replication cohort (DILfrequency; Supplementary Fig. 11e,g - n = 5 patients profiled at 4 timepoints).

5) Circulating CD4+ TFH cells: surface immunostaining in whole-blood flow cytometry data collected from the 18 DILfrequency participants treated with the 3-day interval dosing schedule (profiled at each visit; Supplementary Fig. 12b); and intracellular immunostaining in the multiple dosing replication cohort (DILfrequency; Supplementary Fig. 12c - n = 5 patients profiled at 4 timepoints).

Randomization

Sample randomization was not possible due to the nature of the experimental design. Flow cytometry was performed on whole-blood samples from the DILfrequency study participants at the time of their visit. Single-cell RNA-sequencing was performed on cryopreserved

PBMC samples collected from the study participants and selected according to the IL-2 schedule.

The effect of covariates on the single-cell multiomics dataset was mitigated by the within-donor comparison (n=13 participants). Post-treatment timepoints (Day 27 and Day 55) were compared from the baseline sample (Day 0) of the same donor. All three assessed visits from single participants were processed in parallel to minimize experimental batch effects. Similarly, for the FACS data, all analyses were performed by comparing the post-treatment visits to the respective baseline visit.

Blinding

Blinding was not possible, as donor information and sample visit were required for batch selection. Samples from the same donor were processed for single-cell RNA-sequencing together to avoid batch effects due to day-to-day variation in cell capture and library preparation. Donor and visit information was blinded from the initial QC and clustering steps of the data analysis.

Reporting for specific materials, systems and methods

We require information from authors about some types of materials, experimental systems and methods used in many studies. Here, indicate whether each material, system or method listed is relevant to your study. If you are not sure if a list item applies to your research, read the appropriate section before selecting a response.

Materials & experimental systems

n/a	Involved in the study
☐	☒ Antibodies
☒	☐ Eukaryotic cell lines
☒	☐ Palaeontology and archaeology
☒	☐ Animals and other organisms
☐	☒ Human research participants
☒	☐ Clinical data
☒	☐ Dual use research of concern

Methods

n/a	Involved in the study
☒	☐ ChIP-seq
☐	☒ Flow cytometry
☒	☐ MRI-based neuroimaging

Antibodies

Antibodies used

Flow cytometry (immunophenotyping): CD25-APC (2A3; BD Biosciences #340907); CD25-APC (M-A251; BD Biosciences #555434); CD127-PE-Cy7 (eBioRDR5; eBioscience #25-1278-42); CD19-BUV737 (HIB19; BD Biosciences #612750); CD3-BV510 (UCHT1; BD Biosciences #563109); CD4-BUV395 (SK3; BD Biosciences #563550); CD4-BUV737 (SK3; BD Biosciences #741829); CXCR5-FITC (J252D4; BioLegend #356914); CXCR3-PercP-Cy5.5 (G025H7; BioLegend #353714); CCR5-AF700 (J418F1; BioLegend #359116); CD8-AF700 (RPA-T8; BioLegend#301028); CD8-APC-Cy7 (RPA-T8; BioLegend #301016); PD1-BV421 (EH12.2H7; BioLegend #329920); CD62L-BV605 (DREG-56; BD Biosciences #562719); CD56-BV711 (HCD56; BioLegend #318336); CD45RA-BV785 (HI100; BioLegend #304140); ICOS-PE (C398.4A; BioLegend #313508); CXCR4-PE-CF594 (12G5; BD Biosciences #562389); TIGIT-PerCP-eFluor710 (MBSA43; eBioscience #46-9500-42); CD161-BV421 (HP-3G10; BioLegend #339914); IL6R-PE (M5; BD Biosciences #551850); HLA-DR-AF700 (L243; BioLegend #307626); CD27-BV605 (O323; BioLegend #302830); CXCR5-PE-Dazzle594 (J252D4; BioLegend #356928); FOXP3-PE (259D; BioLegend #320208); FOXP3-PE-eFluor710 (PCH101; eBioscience #46-5773-82); HELIOS-FITC (22F6; BioLegend #137214); Ki67-BUV395 (B56; BD Biosciences #564071).

Sorting: CD25-PE (M-A251; BD Biosciences #555434); CD127-PE-Cy7 (eBioRDR5; eBioscience #25-1278-42); CD3-BV510 (UCHT1; BD Biosciences #563109); CD4-FITC (OKT4; BioLegend #317408); CD56-BV711 (HCD56; BioLegend #318336); CD8-AF700 (HIT8a; BioLegend #300920).

In vitro Treg suppression Assay: CD4-APC-Cy7 (RPA-T4; BioLegend #300518); CD14-PerCP-Cy5.5 (HCD14; BioLegend #325622); CD19-PacBlue (HIB19; BioLegend #302232); CD25-PE (M-A251; BD Biosciences #555432); CD25-PE (2A3; BD Biosciences #341009); CD127-AF647 (HIL-7R-M21; BD Biosciences #558598).

Single-cell RNA-seq (AbSeq): B7-H2 (CD275) 2D3/B7-H2 (BD Biosciences #940091); BTLA (CD272) J168-540 (BD Biosciences #940105); c-kit (CD117) YB5.B8 (BD Biosciences #940051); CCR4 (CD194) 1G1 (BD Biosciences #940047); CCR5 (CD195) 2D7 (BD Biosciences #940050); CCR6 (CD196) 11A9 (BD Biosciences #940033); CCR7 (CD197) 3D12 (BD Biosciences #940014); CD11b M1/70 (BD Biosciences #940008); CD11c B-ly6 (BD Biosciences #940024); CD16 3G8 (BD Biosciences #940006); CD161 DX12 (BD Biosciences #940070); CD226 DX11 (BD Biosciences #940075); CD28 CD28.2 (BD Biosciences #940017); CD38 HIT2 (BD Biosciences #940013); CD39 TU66 (BD Biosciences #940073); CD4 SK3 (BD Biosciences #940001); CD40 5C3 (BD Biosciences #940049); CD40L (CD154) TRAP1 (BD Biosciences #940053); CD45RA HI100 (BD Biosciences #940011); CD45RO UCHL1 (BD Biosciences #940022); CD49a SR84 (BD Biosciences #940094); CD49b (ITGA2) 12F1 (BD Biosciences #940087); CD49d 9F10 (BD Biosciences #940059); CD62L DREG-56 (BD Biosciences #940041); CD69 FN50 (BD Biosciences #940019); CD8 RPA-T8 (BD Biosciences #940003); CD80 L307.4 (BD Biosciences #940036); CD86 FUN1 (BD Biosciences #940025); CR2 (CD21) B-ly4 (BD Biosciences #940048); CTLA-4 (CD152) BNI3 (BD Biosciences #940034); CXCR3 (CD183) 1C6 (BD Biosciences #940030); CXCR4 (CD184) 12G5 (BD Biosciences #940056); CXCR5 (CD185) RF8B2 (BD Biosciences #); DPP4 (CD26) M-A261 (BD Biosciences #940101); FAS (CD95) DX2 (BD Biosciences #940037); GARP (LRRC32) 7B11 (BD Biosciences #940042940217); GITR (CD357) V27-580 (BD Biosciences #940096); HLA-DR G46-6 (BD Biosciences #940010); ICAM2 (CD102) CBR-IC2/2 (BD Biosciences #940241); ICOS (CD278) DX29 (BD Biosciences #940043); IFNGRA (CD119) GIR-208 (BD Biosciences #940253); IGSF2 (CD101) V7.1 (BD Biosciences #940269); IL-12RB1 (CD212) 2-4E6 (BD Biosciences #940267); IL-15RA (CD215) JM7A4 (BD Biosciences #940290); IL-2RA (CD25) 2A3 (BD Biosciences #940009); IL-2RB (CD122) Mik-β3 (BD Biosciences #940232); IL-6R (CD126) M5 (BD Biosciences #940090); IL-7R (CD127) HIL-7R-M21 (BD Biosciences #940012); ITGAE (CD103) Ber-ACT8 (BD Biosciences #940067); ITGB7 FIB504 (BD Biosciences #940244); KLRD1 (CD94) HP-3D9 (BD Biosciences #940081); LAG-3 T47-530 (BD Biosciences #940080); MCP (CD46) E4.3 (BD Biosciences #940211); NCAM-1 (CD56) NCAM16.2 (BD Biosciences #940007); NKp46 (CD335) 9-E2 (BD Biosciences #940064); NT5E (CD73) AD2 (BD Biosciences #940294); OX40 (CD134)

ACT35 (BD Biosciences #940060); PD-1 (CD279) EH12.1 (BD Biosciences #940015); PDL1 (CD274) MIH1 (BD Biosciences #940035); PDL2 (CD273) MIH18 (BD Biosciences #940071); PECAM1 (CD31) WM59 (BD Biosciences #940254); TIM3 (CD366) 7D3 (BD Biosciences #940066); TNFRSF9 (CD137) 4B4-1 (BD Biosciences #940055); Vd2TCR B6 (BD Biosciences #940297); Vg9TCR B3 (BD Biosciences #940295).

Validation	All antibodies were used accordingly with the manufacturer's instruction. Full list of the antibodies used in this study and respective panel information is provided in Supplementary Data 2.<br><br>Antibody validation statements and relevant peered-reviewed publications using the antibodies can be found on the respective antibody website page, associated with the individual catalogue number provided in Supplementary Data 2.

Human research participants

Policy information about studies involving human research participants

Population characteristics	All baseline characteristics of study participants are summarised in supplementary Data 1.
Recruitment	Participants were recruited from the DILfrequency (ClinicalTrials.gov Identifier: NCT02265809) and DILT1D (ClinicalTrials.gov Identifier: NCT01827735) studies.<br><br>To mitigate self-selection bias in patient recruitment, eligible potential participants interested in participating in the DILfrequency study were invited to attend for an appointment where the chief investigator (CI) or his delegate discussed the study with the participant and provided written informed consent before undergoing any trial-related procedures.
Ethics oversight	East of England - Cambridge East Research Ethics Committee (13/EE/0020 & 14/EE/1057 for the DILT1D and DILfrequency studies, respectively)

Note that full information on the approval of the study protocol must also be provided in the manuscript.

Flow Cytometry

Plots

Confirm that:

- [x] The axis labels state the marker and fluorochrome used (e.g. CD4-FITC).
- [x] The axis scales are clearly visible. Include numbers along axes only for bottom left plot of group (a 'group' is an analysis of identical markers).
- [x] All plots are contour plots with outliers or pseudocolor plots.
- [x] A numerical value for number of cells or percentage (with statistics) is provided.

Methodology

Sample preparation	Flow cytometry was performed on whole-blood of all DILfrequency study participants, within 4 hours of phlebotomy. 150 ul of whole blood were incubated for 45 min at room temperature with fluorochrome-conjugated antibodies (with 50 ul Brilliant stain buffer; BD Biosciences), then red blood cells were lysed using the BD FACS lysing solution according to the manufacturer's instructions.<br><br>Flow cytometry on the follow-up single and multiple dosing cohorts was performed on cryopreserved PBMCs. Cells were thawed at 37ºC, incubated with Fixable Viability Dye eFluor 780 for 15 min at 4°C and stained with fluorochrome-conjugated antibodies against surface-expressed markers for 45 min at 4ºC (with 50 ul Brilliant stain buffer; BD Biosciences). Fixation and permeabilisation was performed using the FOXP3 Fix/Perm Buffer Set (eBioscience) according to the manufacturer's instructions, and cells were then stained with fluorochrome-conjugated antibodies against intracellular markers for 1 h at room temperature (with 50 ul Brilliant stain buffer; BD Biosciences).<br><br>For the single-cell RNA-seq experiments, cryopreserved PBMCs collected from the DILfrequecy participants were thawed at 37ºC and resuspended drop-wise in X-VIVO15 + 1% heat-inactivated, filtered human AB serum. Cells were then washed and stained with Fixable Viability Dye eFluor780 (eBiosciences) for 10 min at room temperature. After further washing cells were then incubated fluorochrome-conjugated antibodies for 30 min at 4ºC (with 50 ul Brilliant stain buffer; BD Biosciences) prior to sorting.
Instrument	BD LSR Fortessa (BD Biosciences) and FACSAria Fusion sorter (BD Biosciences)
Software	FACSDiva v8.0.1 (BD Biosciences; data collection) and FlowJo v10.8.1 (BD; data analysis)
Cell population abundance	The following percentages of the 5 assessed immune cell subsets were sorted from each individual for each time point:<br>CD4+ Tregs = 30%<br>CD4+ Teffs = 25%<br>CD8+ T cells = 25%<br>CD56br NK cells = 12%<br>CD56dim NK cells = 8%.

Post-sort purifications were not directly assessed by FACS (due to sample availability) but were inferred from the single-cell RNA-seq data using the respective oligo-conjugated sample tags, which were used to barcode each sorted subset.

Gating strategy	For all flow cytometry-based experiments, an initial FSC-A/SSC-A gate was delineated to identify the lymphocyte fraction and exclude cell debris. Doublet cells were subsequently excluded using a FSC-A/FSC-W gate followed by a FSC-A/SSC-W gate.<br><br>For cell sorting from cryopreserved PBMCs (single-cell RNA-seq experiments), dead cells were then excluded using the Fixable Viability Dye eFluor780 (eBiosciences), by gating on the negative fraction (APC-Cy7 negative gate).<br><br>All subsequent steps used to delineate all relevant immune subsets are shown in detail on Fig. 1 and Supplementary Fig. 1.

☒ Tick this box to confirm that a figure exemplifying the gating strategy is provided in the Supplementary Information.
